# A proteome-wide atlas of humoral immunity to *Mycobacterium tuberculosis* across the spectrum of disease

**DOI:** 10.3389/fimmu.2026.1810894

**Published:** 2026-06-03

**Authors:** Mehak Z. Khan, Edward B. Irvine, Deniz Cizmeci, Leela R. L. Davies, Arlo Z. Randall, Jozelyn Pablo, Samuel J. Vidal, Ninaad Lasrado, Patricia A. Darrah, Jeff C. Hsiao, Colin Mann, Shreya Nair, Patricia S. Grace, Laura Fontana, Boris Juelg, Dan H. Barouch, Blanca I. Restrepo, Mario Roederer, Robert A. Seder, Robert S. Wallis, Gavin Churchyard, Catherine M. Stein, W. Henry Boom, Joseph J. Campo, Sarah M. Fortune, Galit Alter

**Affiliations:** 1 Ragon Institute of MGH, MIT and Harvard, Cambridge, MA, United States; 2 Department of Immunology and Infectious Diseases, Harvard T.H. Chan School of Public Health, Boston, MA, United States; 3 Department of Biological Engineering, Massachusetts Institute of Technology, Cambridge, MA, United States; 4 Antigen Discovery Inc., Irvine, CA, United States; 5 Beth Israel Deaconess Medical Center and Harvard Medical School, Boston, MA, United States; 6 Vaccine Research Center, National Institute of Allergy and Infectious Diseases (NIAID), National Institutes of Health, Bethesda, MD, United States; 7 School of Public Health, University of Texas Health Science Center at Houston, Brownsville, TX, United States; 8 The Aurum Institute, Johannesburg, South Africa; 9 Department of Medicine, Division of Infectious Diseases, School of Public Health, University of the Witwatersrand, Johannesburg, South Africa; 10 Department of Medicine, Vanderbilt University, Nashville, TN, United States; 11 Department of Medicine, Case Western Reserve University, Cleveland, OH, United States

**Keywords:** antibodies, antigens, microarray, *Mycobacterium tuberculosis*, proteome, serology, vaccines

## Abstract

Exposure to *Mycobacterium tuberculosis* (*Mtb*) leads to a spectrum of outcomes, from latent infection to active disease, but limited insight into protective immune correlates has hampered vaccine development. Using a proteome-wide *Mtb* microarray, we mapped antibody specificities across individuals with varying *Mtb* exposure, including humans with controlled latent tuberculosis infection, uncontrolled active TB, or that resist IGRA conversion as well as non-human primates with near sterilized *Mtb* infection following intravenous BCG. While current TB vaccine antigens were poorly immunogenic across most populations, striking overlap in antibody binding was observed to surface and secreted proteins, all associated with enhanced antibody functions and some also robustly targeted by T cells. Collectively, these data provide an atlas of antibody protein binding across the spectrum of Mtb infection, and further point to a handful of promising Mtb protein candidates to guide the design of antibody-based TB interventions aimed at mitigating disease globally.

## Introduction

Tuberculosis (TB) caused an estimated 1.23 million deaths in 2024 (1). Despite effective antibiotic regimens, *Mycobacterium tuberculosis* (*Mtb*) continues to pose a major global health threat, exacerbated by diagnostic challenges, limited vaccine efficacy, and the emergence of rifampicin-resistant strains—now designated among Priority 1 AMR pathogens by the World Health Organization. While most *Mtb*-exposed individuals develop latent TB infection (LTBI), a small subset, known as TB ‘resisters’ (RSTRs), remain persistently negative on standard diagnostics and never develop disease despite repeated exposure (2–6) (7). Unfortunately, the immunological basis of this resistance remains incompletely understood.

T cells are critical for TB control (8–10) and have long guided vaccine (11–13) and diagnostic development (14). However, T cell-based diagnostics such as tuberculin skin test (TST) and interferon-gamma release assay (IGRA) poorly predict disease progression and fail to capture the RSTR phenotype (14, 15). Furthermore, vaccine candidates designed to elicit strong T cell responses have demonstrated limited efficacy in clinical trials (11, 16). Thus, identifying reliable immune correlates of protection remains a high priority for the development of new TB interventions.

Although antibodies are a cornerstone of diagnostics and therapeutics for many infectious diseases, their role in TB has been historically overlooked. Despite our emerging appreciation for a role for antibodies as a biomarker of disease state and in *Mtb* control (16, 17), little is known about the specific targets of antibody responses to *Mtb*. Antibodies may contribute directly to *Mtb* control or may simply reflect CD4^+^ T cell activity, which is essential for class switching (18–22). Therefore, the presence of Mtb-specific antibody responses could offer insight into both humoral and T cell-mediated immunity.

Studies of the humoral response to *Mtb* have often been limited to a handful of antigenic targets encoded by the bacterium. Yet, several studies in recent years have explored antibody responses elicited during *Mtb* infection to hundreds (23, 24), and even thousands of antigens (25, 26). This work has identified numerous *Mtb* proteins that elicit a robust IgG antibody response in the setting of active TB (ATB) (23–26). However, these studies did not define whether unique IgG reactivity profiles may selectively arise in the setting of natural- or vaccine-induced TB control, rendering antibody binding profiles elicited in the setting of protective immunity unknown for a majority of the *Mtb* proteome.

In this study, we applied a comprehensive *Mtb* proteome-wide microarray (~4,000 proteins) to map IgG responses across individuals with varying clinical outcomes, from ATB to LTBI and RSTRs, as well as non-human primates immunized with either standard intradermal (ID) or protective intravenous (IV) BCG (**Supplementary Figure 1**). By analyzing IgG binding as both a proxy for CD4^+^ T cell engagement (27) and a possible mediator of antimicrobial activity (17), we identified antigenic signatures unique to each cohort. Notably, a shared subset of antibody targets was enriched in LTBI, RSTRs and IV-BCG-immunized NHPs—populations with enhanced control over *Mtb* infection and validated biophysical and functional antibody responses to selected antigens. Thus, this work provides a high-resolution atlas of humoral immune responses across the spectrum of TB exposure and disease. While the precise role of antibodies—whether mechanistic or surrogate—remains to be clarified, this study provides a step in identifying vulnerable *Mtb* antigens that may inform the design of next-generation TB vaccines, therapeutics, and diagnostics.

## Results

### Individuals with ATB and LTBI exhibit distinct IgG binding profiles

**Figure 1:**
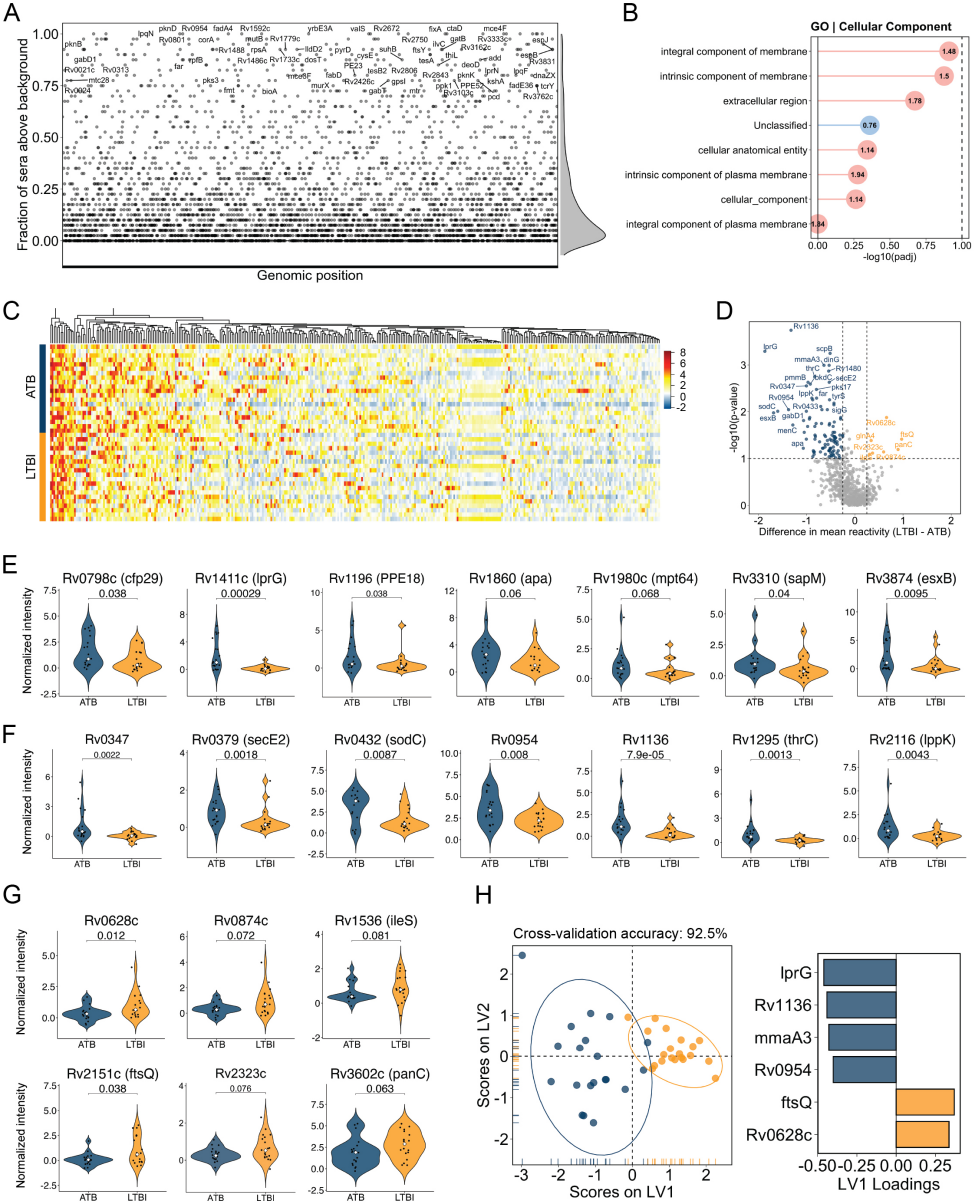
Distinct IgG binding profiles in ATB and LTBI subjects. **(A)** Manhattan plot showing the fraction (percentage) of serum samples with a median normalized intensity score 1.25-fold over the IVTT only background for each antigen. **(B)** Gene Ontology (GO) enrichment analysis. Subset of antigens with a normalized intensity score 1.25-fold over background in over 75% of individuals in the were used as the input for GO analysis. Positively enriched gene sets are pink, negatively enriched gene sets are light blue. Points are labeled with the fold enrichment. GO terms greater than the dashed line at a Benjamini-Hochberg adjusted p-value of 0.1 were considered significant. **(C)** Heatmap summary of normalized IgG intensity of each antigen above the reactivity threshold (median normalized binding intensity at least 1.25-fold over background in at least 1 group). Rows segregated by group. Columns hierarchically clustered. **(D)** Volcano plot showing differential reactivity analysis. Antigens with |μ_LTBIintensity_ – μ_ATBintensity_ | > 0.25 (vertical dashed lines) and unadjusted Mann-Whitney p < 0.1 (horizontal dashed line) were considered differentially reactive and are colored. ATB-enriched antigens (blue). LTBI-enriched antigens (yellow). **(E–G)** Violin plots of differentially reactive antigens in, **(E)** ATB previously identified, **(F)** ATB newly identified, **(G)** LTBI newly identified. Mann-Whitney p-value shown. **(H)** LASSO PLS-DA analysis distinguishing ATB from LTBI subjects by IgG binding profile. Score plot (left). Ellipses show 95% confidence intervals for each population. Latent variable (LV) 1 loadings plot of LASSO-selected antigens (right).

Serum samples were collected from twenty adult human immunodeficiency virus (HIV) seronegative individuals with LTBI and twenty with ATB at the United States-Mexico border (**Supplementary Figure 1**) (28). Antigen specific class switched IgG binding intensity, marking the presence of an antibody response or T cell help, was quantified on an *Mtb* protein microarray (25, 26). The microarray contained polypeptides produced in a cell free *in vitro* transcription/translation (IVTT) system, covering ~95% of *Mtb* open reading frames, and allowing the query of antibody reactivity across nearly the whole *Mtb* proteome (25, 26). After data pre-processing, normalized intensity scores representing IgG titers to each of 3963 antigens were obtained for each sample. Out of the 3963 antigens, only 650 had a normalized intensity score above our empirically determined reactivity threshold, and thus had a median binding intensity 1.25-fold over background in at least one group (16.42%), pointing to a considerable segment of the *Mtb* proteome which may poorly elicit peripheral IgG responses during ATB and/or LTBI. Antigens that consistently elicited IgG responses above background independent of group included: Rv0044c, Rv1323 (FadA4), Rv3043c (CtaD), Rv3494c (Mce4F), and Rv3879c (EspK) (**Figure 1A** and **S2A**), representing highly immunogenic antigens indiscriminately targeted by antibodies in individuals with ATB and LTBI. To gain deeper insights on the nature of the immunogenic proteins, we performed Gene Ontology (GO) enrichment analysis using the group of antigens with a normalized intensity score 1.25-fold over background in over 75% of individuals in the study population. Cell membrane and extracellular protein GO terms exhibited a trend towards overrepresentation in the gene set, likely reflecting the redundancy in many surface and secreted antigens involved in bacterial defense, yet none of the GO terms reached statistical significance following multiple testing correction (**Figure 1B****).**

We next aimed to identify the select antigens uniquely targeted during ATB and LTBI respectively by comparing the normalized IgG binding intensity to each antigen above our reactivity threshold across disease states (**Figure 1C**). 109 antigens were differentially targeted between the groups (|μ_LTBIintensity_ – μ_ATBintensity_| > 0.25 and unadjusted Mann-Whitney p-value < 0.1) (**Figure 1D**). Consistent with previous reports of higher antibody titers in ATB as compared to LTBI subjects, 102 of these differentially reactive antigens exhibited higher IgG binding intensities in the setting of ATB, with only 7 antigens targeted selectively in the LTBI group (**Figure 1D**). Several of the differentially targeted antigens enriched in ATB group overlapped with those found in previous studies, including: Rv0798c (Cfp29) (29), Rv1196 (PPE18) (29), Rv1411c (LprG) (25, 26), Rv1860(Apa) (23, 25, 26, 29, 30), Rv1980c (Mpt64) (25, 26, 30), Rv3310 (SapM) (23), and Rv3874 (EsxB) (23, 25, 26, 29, 30) (**Figures 1D, E**). Additionally, several novel antigens were targeted more robustly by IgG responses in the setting of ATB such as: Rv0347, Rv0379 (SecE2), Rv0432 (SodC), Rv0954, and Rv1136 (**Figures 1D, F**). Likewise, we identified antigens such as Rv0628c, Rv2151c (FtsQ), Rv2323c, Rv2860c (GlnA4), and Rv3602c (PanC) as being more strongly targeted during LTBI (**Figures 1D, G**, **Supplementary Table 1****).**

Next, to define a minimal humoral reactivity profile able to differentiate ATB from LTBI, we took a computational approach employing least absolute shrinkage and selection operator (LASSO) feature selection to reduce the number of features and reduce the likelihood of overfitting, followed by partial least-squares discriminant analysis (PLS-DA) (31, 32) to visualize and evaluate group separation. Notably, IgG binding intensity to only 6 LASSO-selected proteins allowed the separation of ATB from LTBI subjects with over 90% accuracy (**Figure 1H**). Antigens identified during univariate analyses to be enriched in the IgG responses of individuals with ATB such as Rv1136 and Rv1411c (LprG), and in the IgG responses of individuals with LTBI such as Rv0628c and Rv2151c (FtsQ), drove separation between the groups (**Figure 1H**).

Finally, we performed protein set enrichment analysis to elucidate the classes of proteins enriched in the IgG response patterns of individuals with ATB and LTBI. Each protein in the array was ranked by a significance score integrating both statistical and magnitude difference in normalized intensity between individuals with ATB and LTBI (Materials and Methods). The overrepresentation of *Mtb* protein sets at the top (LTBI-enriched) or bottom (ATB-enriched) of the ranked list was then determined by gene set enrichment analysis (33, 34). However, no protein classes were significantly enriched when comparing ATB with LTBI individuals (**Supplementary Figure 2B**). Together, these data demonstrate that subjects with ATB and LTBI can be identified by their class switched IgG reactivity profiles to select *Mtb* antigens. While IgG titers to most antigens were higher in the ATB group, notably, several antigens were targeted more strongly by IgG responses in individuals with LTBI that contained *Mtb* infection.

### RSTR and LTBI subjects exhibit distinct IgG binding profiles

**Figure 2:**
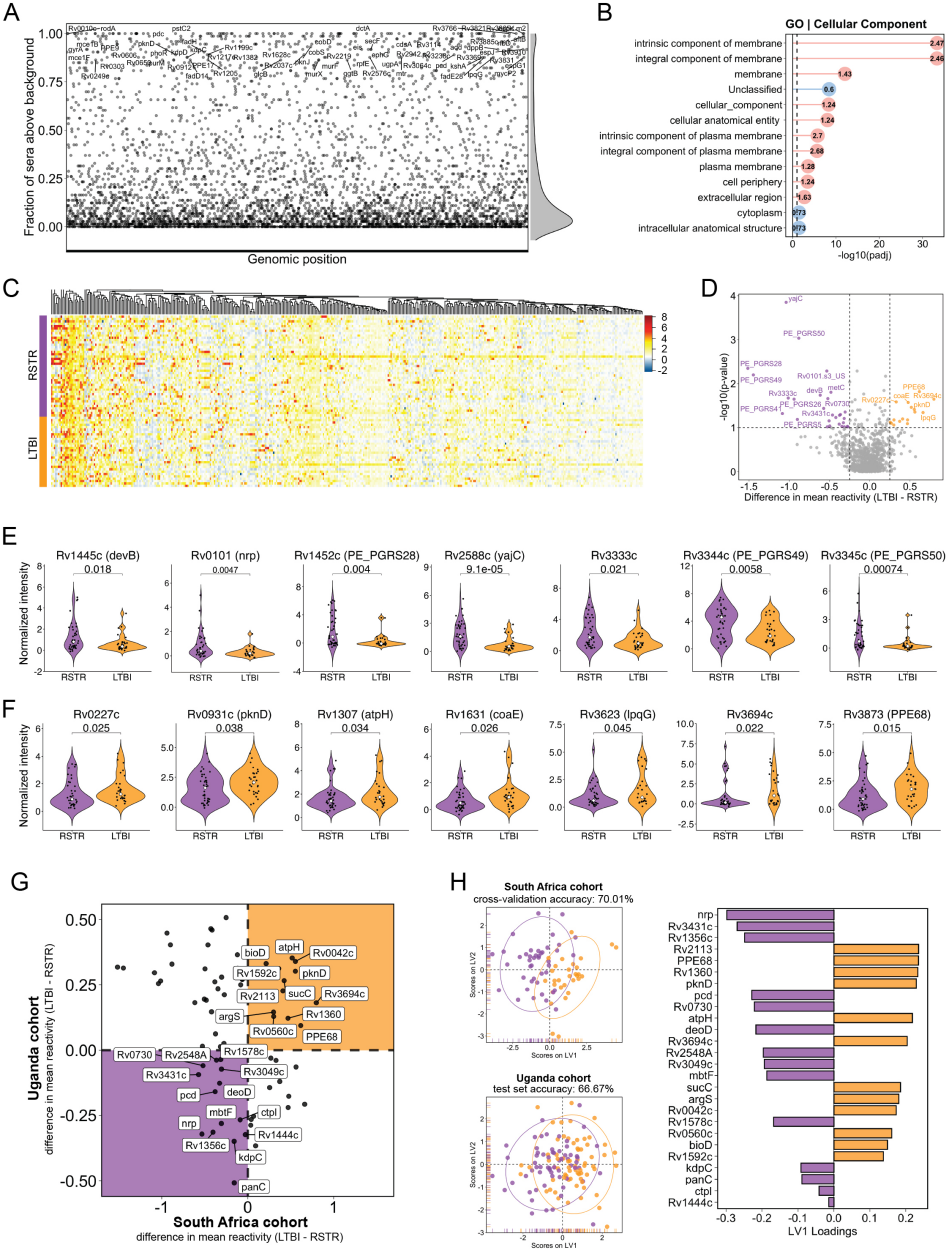
Converging IgG binding patterns across distinct RSTR and LTBI cohorts. **(A)** Manhattan plot showing the fraction (percentage) of serum samples with a median normalized intensity score 1.25-fold over the IVTT only background for each antigen. **(B)** GO enrichment analysis. Antigens with a normalized intensity score 1.25-fold over background in over 75% of individuals in the were used as the input for GO analysis. Positively enriched gene sets are pink, negatively enriched gene sets are light blue. Points are labeled with the fold enrichment. GO terms greater than the dashed line at a Benjamini-Hochberg adjusted p-value of 0.1 were considered significant. **(C)** Heatmap summary of normalized IgG intensity of each antigen above the reactivity threshold. Rows segregated by group. Columns hierarchically clustered. **(D)** Volcano plot showing differential reactivity analysis. Antigens with |μ_LTBIintensity_ – μ_RSTRintensity_ | > 0.25 (vertical dashed lines) and unadjusted Mann-Whitney p < 0.1 (horizontal dashed line) were considered differentially reactive and are colored. RSTR-enriched antigens (purple). LTBI-enriched antigens (yellow). **(E, F)** Violin plots of differentially reactive antigens in, **(E)** LTBI, **(F)** RSTRs. Mann-Whitney p-value shown. **(G)** Scatter plot of differentially reactive antigens identified in either the South African cohort or the Ugandan cohort. Difference in mean reactivity between LTBI subjects and RSTRs in the South African (x-axis) and Ugandan (y-axis) cohort are plotted. Antigens in colored quadrants are enriched in RSTRs (purple, bottom left), and LTBI subjects (yellow, top right) across both cohorts. **(H)** PLS-DA analysis. Model was trained on South African cohort using select antigens showing consistent patterns across South African and Ugandan cohorts. South African score plot (top left). Ellipses show 95% confidence intervals for each population. Ugandan cohort was used as held-out test set to evaluate model. Projection of Uganda cohort into latent variable space of the trained model (bottom left). Loadings plot shows the contribution of each antigen to LV1 (right).

A fraction of highly *Mtb*-exposed individuals, referred to as RSTRs, persistently test negative using the standard TB diagnostics – TST and IGRA – despite consistently high levels of exposure to *Mtb* (2–6). Despite the lack of TB diagnostic positivity, RSTRs possess robust *Mtb*-specific humoral immune responses linked to unique *Mtb*-specific T cell immune responses that secrete several cytokines in the absence of interferon-γ (IFNγ) (7), pointing to the possibility that RSTRs may represent an additional clinical phenotype of *Mtb* control, with limited evidence of progression to TB. Because antibody Fc-profiles in RSTRs differed from those elicited in LTBI (7), we hypothesized that RSTRs may also raise IgG responses to a unique collection of antigens, and thus provide additional insights into unique immunological targets across the *Mtb* proteome. Serum samples from RSTR (n=43) and LTBI (n=30) subjects were collected from HIV negative gold miners in South Africa who had worked at least 15 years in the mining industry (**Supplementary Figure 1**) (35, 36). These individuals were subjected to extremely high levels of *Mtb* exposure due to their confined working and living conditions (3, 37, 38). Subjects with persistent negative TST and IGRA results were defined as RSTRs. Antibody profiling across the two groups revealed that 849 of the total 4187 proteins exhibited a normalized binding intensity over our reactivity threshold, pointing to detectable IgG responses against 20.28% of the *Mtb* proteome. Antigens targeted most widely by IgG responses, agnostic of group, included: Rv0010c, Rv0171 (Mce1C), Rv0583c (LpqN), Rv2100, and Rv2620c (**Figures 2A** and **S3A**). Gene Ontology enrichment analysis using antigens with a normalized intensity score 1.25-fold over background in over 75% of individuals in the study population, showed that antibody binding intensity to proteins in the cell membrane and extracellular region were significantly positively enriched, and thus strongly targeted by IgG responses in RSTRs and subjects with LTBI (**Figure 2B**). Conversely, cytoplasmic proteins were significantly negatively enriched (**Figure 2B**), suggesting a limited immunogenicity of intracellular *Mtb* proteins.

To next define whether distinct antigens were targeted by IgG responses in the RSTRs and LTBI subjects from South Africa, we compared the difference in normalized IgG binding intensity to each protein above our reactivity threshold between groups (|μ_LTBIintensity_ – μ_RSTRintensity_| > 0.25 and unadjusted Mann-Whitney p-value < 0.1) (**Figure 2C**). Several antigens showed increased antibody binding in the IgG reactivity profile of RSTR subjects, such as: Rv1445c (DevB), Rv1452c (PE_PGRS28), Rv2588c (YajC), Rv3344c (PE_PGRS49), and Rv3345c (PE_PGRS50) (**Figure 2D, E**). Several antigens were more robustly targeted by IgG in individuals with LTBI including: Rv1631 (CoaE), Rv1307 (AtpH), Rv3873 (PPE68), Rv0227c, and Rv3694c (**Figures 2D, F**). Of note, the number, magnitude, and significance of the differentially targeted antigens was increased in the RSTR group as compared to the LTBI group (**Figure 2D**; **Supplementary Table 1**). By LASSO PLS-DA, a group of just 9 RSTR- and 4 LTBI-defining antigens were sufficient to discriminate RSTR and LTBI subjects with approximately 86% classification accuracy (**Supplementary Figure 3B**).

Finally, we queried the enrichment of specific protein classes in the antigens targeted by IgG responses in LTBI and RSTR individuals from South Africa. While LTBI subjects were broadly enriched in IgG targeting virulence related proteins and the PPE family (defined by a conserved N-terminal Proline-Proline-Glutamic acid (PPE) motif), RSTRs exhibited enriched binding to the polymorphic GC-rich-repetitive sequence (PGRS) subfamily of PPE proteins (39, 40), suggesting a distinct IgG humoral profile diverted towards this unique subfamily of *Mtb* virulence factors (**Supplementary Figure 3C**). Collectively, these data indicate that although RSTRs remain invisible to our current arsenal of TB diagnostics, RSTRs elicit detectable IgG responses to a distinct subset of *Mtb* proteins than do individuals with LTBI.

### Converging IgG binding profiles across exposure contexts in RSTRs and LTBI

We next compared IgG reactivity in the South African miner RSTR-LTBI cohort to that of RSTRs and LTBI subjects from a Ugandan household study to determine the impact of differential geography and exposure on IgG binding profiles in these populations (4, 5, 7, 35). For the Ugandan cohort, HIV negative household contacts of pulmonary TB cases were followed prospectively for up to 2 years and subjected to serial TST/IGRA and clinical assessment (4, 5, 7, 35). Differential IgG reactivity analysis across Ugandan RSTR and LTBI subjects identified 10 antigens that were preferentially targeted in RSTRs and 17 antigens that were targeted largely by LTBI cases (|μ_LTBIintensity_ – μ_RSTRintensity_| > 0.25 and unadjusted Mann-Whitney p-value < 0.1) (**Supplementary Figure 4A**). However, like the South African cohort, RSTRs and LTBI subjects from Uganda could be accurately separated by LASSO PLS-DA modeling, with a cross-validation accuracy of approximately 82% (**Supplementary Figure 4B**). 28 antigens were required for accurate classification (**Supplementary Figure 4C**), pointing to more nuanced differences in IgG binding profiles across the RSTRs LTBI subjects in this household contact cohort. We next compared responses across RSTR/LTBI cohorts from South Africa and Uganda, to define potential common targets (**Figure 2G**). Strikingly, 14 antigens were consistently selectively targeted in the IgG responses of South African and Ugandan RSTRs including: Rv0101 (Nrp), Rv1031 (KdpC), Rv1356c, Rv2379c (MbtF), and Rv3602c (PanC) (**Figure 2G** and **S5A**). Likewise, 12 antigens including: Rv0931c (PknD), Rv1292 (ArgS), Rv1307 (AtpH), Rv1592c, and Rv3694c were consistently preferentially enriched in the IgG responses of subjects with LTBI in the South African and Ugandan cohorts (**Figure 2G** and **S5B**).

Leveraging this intriguing subset of antigens convergently targeted by the peripheral IgG responses of each clinical group, we next aimed to determine whether a single multivariate model would allow the accurate classification of RSTRs and LTBI. The resulting cross-validation accuracy on the South African cohort was 70.01%, and when model performance was evaluated in the held-out Ugandan test set, the classification accuracy decreased only slightly to 66.67%, pointing to the robustness of the model even on unseen data (**Figure 2H**). While this held-out performance was modest, the limited drop in accuracy across cohorts suggests that a subset of LTBI- and RSTR-associated antigens may be consistently targeted by IgG responses across geographically and epidemiologically distinct populations.

### IV BCG drives lung-localized IgG responses specific to a unique collection of antigens

**Figure 3:**
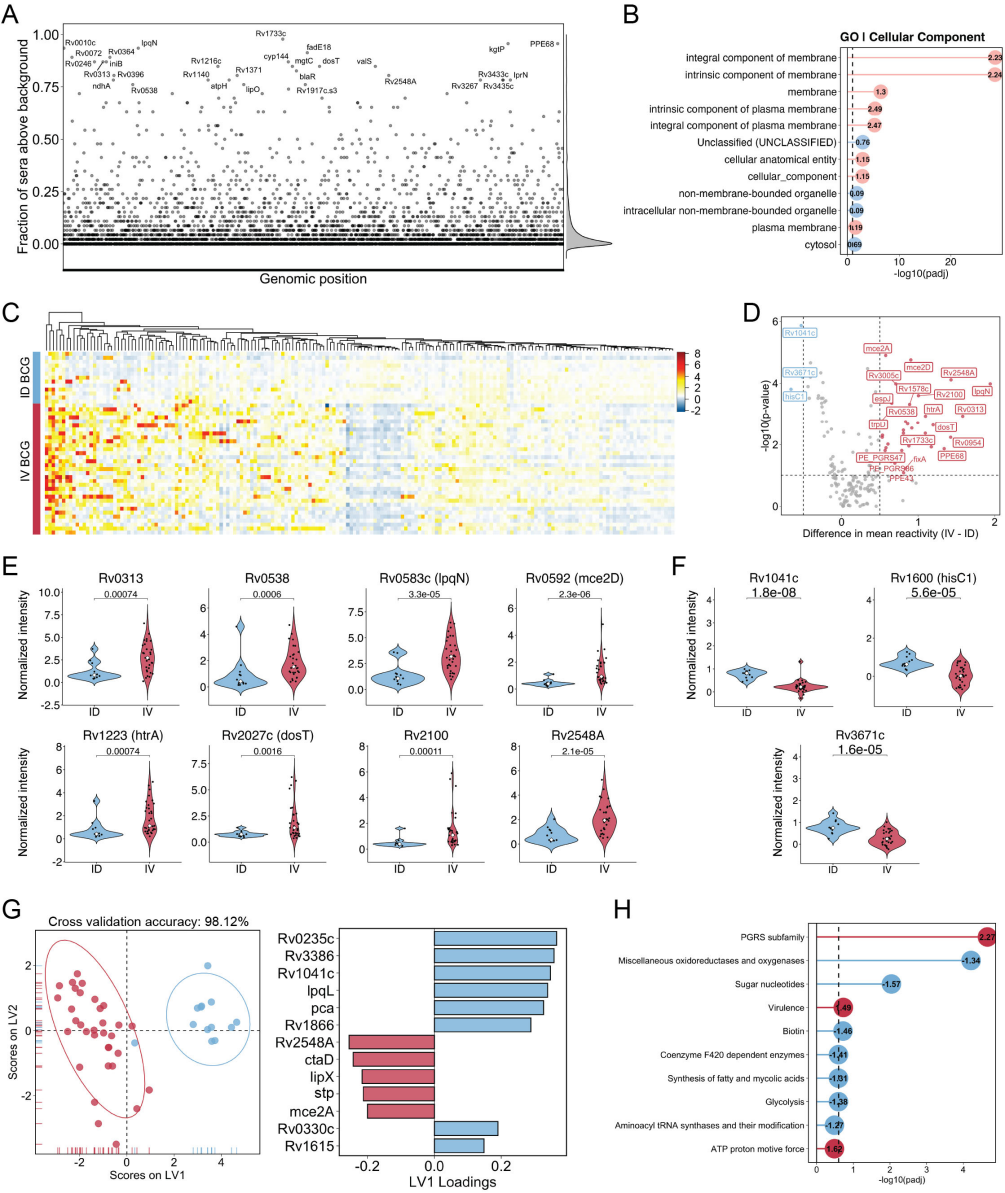
IV BCG drives lung-localized IgG responses specific to a unique collection of antigens. **(A)** Manhattan plot showing the fraction (percentage) of BAL samples with a median normalized intensity score 1.25-fold over the IVTT only background for each antigen. **(B)** GO enrichment analysis. Antigens with a normalized intensity score 1.25-fold over background in over 75% of individuals in the were used as the input for GO analysis. Positively enriched gene sets are pink, negatively enriched gene sets are light blue. Points are labeled with the fold enrichment. GO terms greater than the dashed line at a Benjamini-Hochberg adjusted p-value of 0.1 were considered significant. **(C)** Heatmap summary of normalized IgG intensity of each antigen above the reactivity threshold. Rows segregated by group. Columns hierarchically clustered. **(D)** Volcano plot showing differential reactivity analysis. Antigens with | μ_IVBCGintensity_ – μ_IDBCGintensity_ | > 0.5 (vertical dashed lines) and Benjamini-Hochberg adjusted Mann-Whitney p < 0.1 were considered differentially reactive and are colored. Dashed line at p < 0.1. ID BCG-enriched antigens (light blue). IV BCG-enriched antigens (red). **(E, F)** Violin plots of differentially reactive antigens in **(E)** IV BCG, and **(F)** ID BCG. Mann-Whitney p-value shown. **(G)** LASSO PLS-DA analysis distinguishing IV BCG from ID BCG vaccinated macaques by IgG binding profile. Score plot (left). Ellipses show 95% confidence intervals for each population. LV1 loadings plot of LASSO-selected antigens (right). **(H)** Protein set enrichment analysis across vaccination group using Sanger gene sets. Gene sets enriched in IV BCG are red, gene sets enriched in ID BCG are light blue. Points are labeled with the normalized enrichment score. Gene sets greater than the dashed line at a Benjamini-Hochberg adjusted p-value of 0.25 were considered significant.

While RSTRs and LTBI subjects represent potential states of differential *Mtb* exposure, containment, or even cure, recent advances in TB vaccine development have begun to highlight the potential to induce immunity that confers high-level protection against infection. Specifically, the delivery of bacille Calmette-Guérin (BCG) intravenously affords near-sterilizing immunity in rhesus macaques challenged with *Mtb*. A hallmark of the immune protection afforded by IV BCG was the robust cell-mediated and humoral immunity triggered in the lungs (41, 42). However, whether these uniquely expanded lung immune responses targeted a unique repertoire of *Mtb* antigens remained unclear. Thus, IgG binding profiles in bronchoalveolar lavage fluid (BAL) samples collected from the lungs of rhesus macaques that received standard ID BCG and exhibited poor control of *Mtb* (n=13), were compared to the IgG binding profiles present in the BAL of animals that received IV BCG (n=33). 760 of the 4,187 antigens evaluated showed a normalized IgG binding intensity above our predetermined reactivity threshold in either of the rhesus macaque groups, representing 18.15% of the *Mtb* proteome. Antigens targeted frequently by IgG responses across the entire cohort, independent of group, included: Rv0010c, Rv0072, Rv0583c (lpqN), Rv1733c, and Rv3476c (kgtP) (**Figure 3A** and **S6**). Further, Gene Ontology enrichment analysis revealed that antigens targeted by IgG in the BAL of BCG vaccinated rhesus macaques were significantly positively enriched in cell membrane proteins (**Figure 3B**).

BAL IgG reactivity, potentially marking antibody specificities that may act directly to control the bacteria or linked to anti-microbial CD4 T cell immune responses, both that have been linked to protection against *Mtb* (42, 43), was compared between ID and IV BCG immunized macaques (|μ_IVBCGintensity_ – μ_IDBCGintensity_| > 0.5 and Benjamini-Hochberg adjusted Mann-Whitney p-value < 0.1). Several antigens were targeted more strongly by IgG in macaques receiving protective IV BCG vaccination, such as: Rv0313, Rv0583c (LpqN), Rv0592 (Mce2D), Rv2027c (DosT), and Rv2548A (**Figures 3C–E**). Many antigens remained significantly enriched in the IV BCG group following multiple testing correction (**Figures 3D, E**). In contrast, only Rv1041c, Rv1600 (HisC1), Rv3220c, and Rv3671c were enriched in the ID BCG group following multiple testing correction (**Figures 3D, F**; **Supplementary Table 1**). As expected, given these univariate differences, the two groups of macaques could be accurately resolved using only a small number of LASSO-selected antigens via PLS-DA (**Figure 3G**). Moreover, protein set enrichment analysis pointed to enhanced reactivity to virulence-related proteins in BAL IgG from IV BCG vaccinated macaques, including enhanced immunity to the PGRS subfamily (**Figure 3H**). Together, these data demonstrate that protective IV BCG immunization drives increased IgG titers to a selective set of *Mtb* virulence factors in the lungs.

### Antigens used in TB vaccine development are poorly immunogenic in protected populations

**Figure 4:**
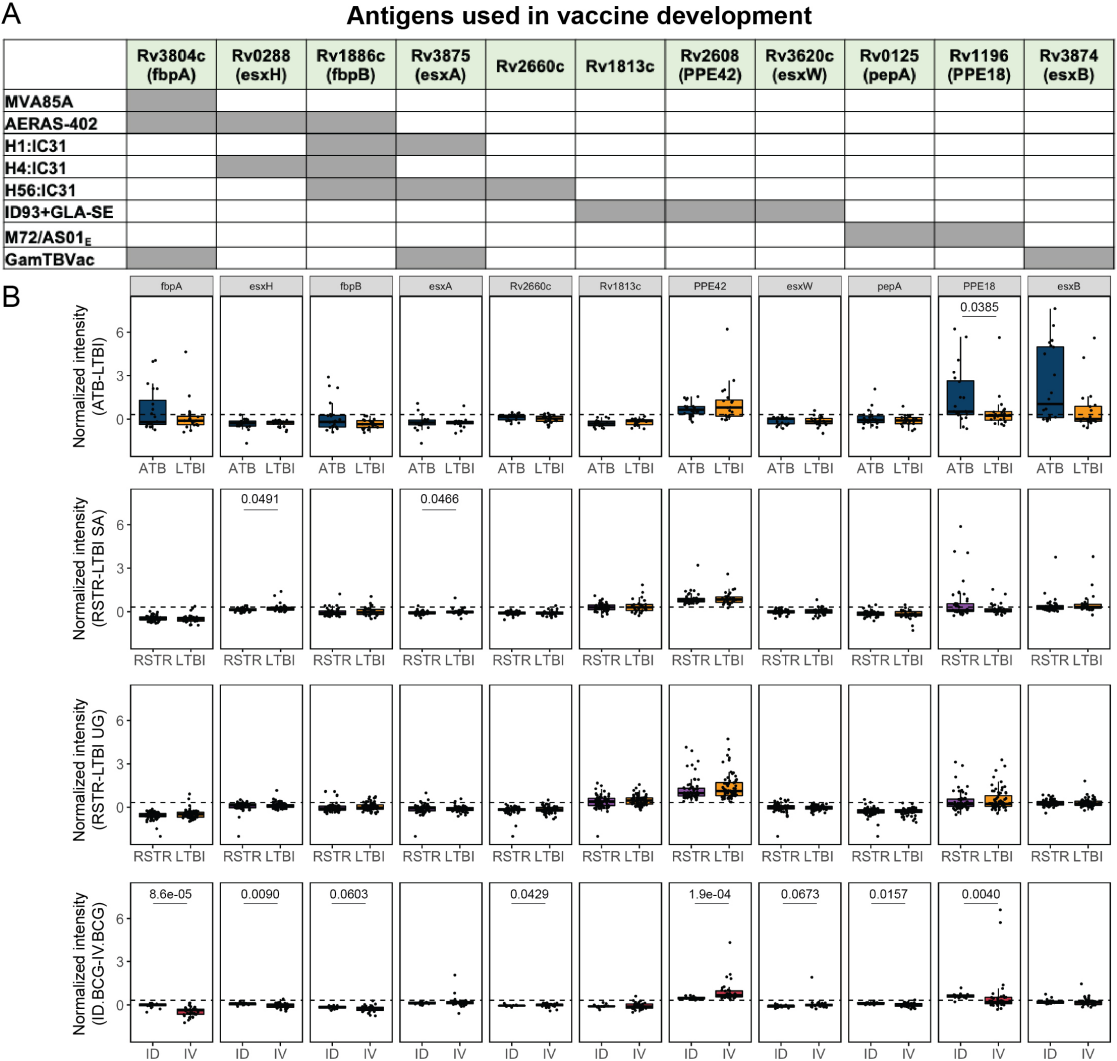
Antigens used in TB vaccine design are poorly recognized by antibodies in protected populations. **(A)** Viral vector and subunit TB vaccines are shown across the rows in the top heatmap, and vaccine antigens included in the vaccines are shown in the columns. Grey boxes signify that a particular antigen was present in the respective vaccine. **(B)** Box plots below the chart show the normalized IgG binding intensity to the TB vaccine antigens for each cohort. Antibody responses by cohort comparisons are shown from top to bottom: row 1 shows ATB-LTBI, row 2 shows South Africa RSTR-LTBI, row 3 shows Uganda RSTR-LTBI, and row 4 shows ID.BCG-IV.BCG vaccinated rhesus macaques. Box plots of vaccine antigen panel follow the same column alignment by antigen as the chart above. The dashed line shows the reactivity threshold (normalized intensity = log_2_(1.25)). Mann-Whitney, p-values < 0.1 are shown.

While several emerging TB vaccines have begun to show partial protection against *Mtb* infection or progression to TB in humans (12, 13, 44), TB vaccine design has been guided nearly exclusively with the goal of optimizing cell-mediated immunity, whereas antibody-informed approaches have not been considered (16). However, while the phase 2b helper T cell-directed MVA-85A TB vaccine trial was unsuccessful, *post-hoc* analysis of correlates of immunity from the trial found Ag85A-specific antibody titers to be associated with reduced risk of TB disease (45). Moreover, given our emerging appreciation for the *in vivo* restrictive activity of antibodies (46–51), it is plausible that *Mtb*-specific antibodies may work in concert with cell-mediated immunity to promote maximal protection against *Mtb*. Thus, to begin to define whether current vaccine targets represent promising antibody targets, we next explored IgG reactivity used in TB viral vector and subunit vaccines in the clinical pipeline (**Figure 4A**). These included the MVA85A (11) and AERAS-402 (52) viral vector vaccines, and the H1:IC31 (53), H4:IC31 (44, 54), H56:IC31 (54), ID93+GLA-SE (55), GamTBVac (56), and M72/AS01_E_ (12, 13) protein subunit vaccines (**Figure 4A**).

Strikingly, 8 of the 11 antigens used in clinical TB vaccine design showed an antibody binding level near or below our reactivity threshold (1.25-fold over background) across each the *Mtb* exposed or vaccinated populations, while only 3 of the 11 antigens displayed notable binding intensity over this threshold (**Figure 4B**). First, IgG binding to Rv2608 (PPE42), from the ID93+GLA-SE vaccine (55), was detectable across each of the populations (**Figure 4B**). Binding intensity to Rv1196 (PPE18), from the M72/AS01_E_ vaccine (12, 13), was significantly higher in ATB compared to LTBI, and in ID BCG compared to IV BCG, but was below our reactivity threshold for each of the protected populations: individuals with LTBI, RSTRs, and IV BCG vaccinated macaques (**Figure 4B**). Finally, antibody binding to Rv3874 (EsxB), from the GamTBVac vaccine (56), was increased in ATB as compared to LTBI, but was negligible across the remaining populations (**Figure 4B**). These results suggest that the antigens utilized in viral vector and subunit TB vaccine candidates in the clinical pipeline are poor drivers of humoral immunity in the studied cohorts, and while responses to a handful of these antigens were present in individuals with ATB, IgG binding to these antigens was particularly low in the setting of natural- and vaccine-induced control of *Mtb*. Together, these data raise the possibility that alternate sets of antigens, which may drive both cellular and humoral immunogenicity, could represent promising targets for next-generation vaccine development clinically.

### Converging IgG responses

**Figure 5:**
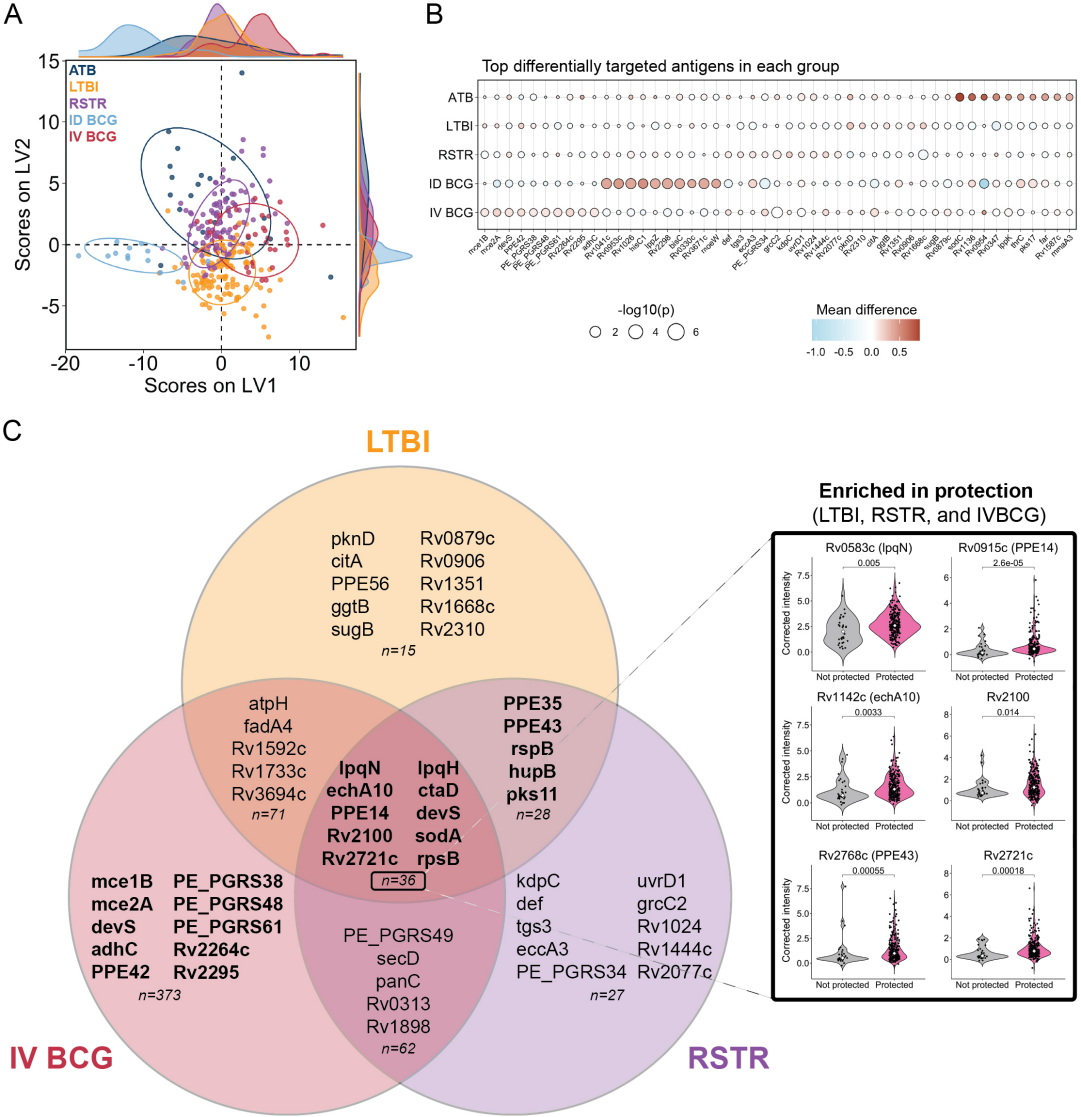
IgG response overlap across cohorts. **(A)** The PLS-DA shows the combined, batch-corrected dataset for all 5 tested populations. Ellipses show 80% confidence intervals for each population. **(B)** The dot plot shows the top differentially targeted antigens in each group. Mann-Whitney one versus all. Dot darkness represents the magnitude of the mean difference. Dot size represents the significance. **(C)** The venn diagram summarizes the overlap of top targeted antigens across the “protected” populations (LTBI, RSTR, and IV BCG). For each region of the Venn diagram, Mann-Whitney U tests were performed to assess differential IgG binding intensity to each antigen using a one versus all approach, comparing that region to the remaining populations. Selected antigens with Mann-Whitney p-value < 0.1 are shown in each region of the Venn diagram. The number of total antigens in each region is indicated. Bolded antigens were significant following Benjamini-Hochberg multiple testing correction. Batch corrected intensity values of select antigens, that were enriched across all the “protected” populations from the center of the Venn diagram, are shown in violin plots (right). Mann-Whitney p-values are shown.

We next aimed to define whether a particular set of antigens may be targeted in the context of distinct states of “controlled” TB. Thus, the datasets were integrated following a harmonization procedure, first restricting analyses to antigens assayed across all cohorts and then removing batch-related variation through linear modeling commonly used for batch correction of bulk RNA-seq data (**Supplementary Figures 7A, B**) (57). From these data, only antigens above background in at least one population were retained in the integrated, batch-corrected dataset (n=1267). To initially capture the overall similarities in IgG reactivity profiles across the groups, we projected the integrated, batch-corrected data into low dimensional space using PLS-DA (**Figure 5A**). The IgG binding profile of BAL from protected, IV BCG immunized macaques overlapped with that of sera from both human RSTR and LTBI profiles, suggesting convergent antigen recognition across these populations (**Figure 5A**). Interestingly, ATB patients were diffusely spread, pointing to considerable heterogeneity in binding profiles in this group (**Figure 5A**). The ID BCG immunized macaque localized separately from the other groups, a pattern that likely reflects the relatively weak BAL IgG responses observed following standard BCG vaccination (**Figure 5A**).

We next identified a number of antigens that were distinctively targeted within individual groups (**Figure 5B**). Group-enriched antigens were observed in ATB, LTBI, RSTR, ID BCG, and IV BCG samples using a one-versus-all comparison (unadjusted Mann-Whitney p-value < 0.1) (**Figures 5B, C****).** Notably, several antigens were selectively targeted by BAL IgG responses in IV BCG-immunized macaques even after multiple testing correction, including Rv0589 (Mce2A), Rv2162c (PE_PGRS38), Rv2264c, Rv2608 (PPE42), and Rv3132c (DevS) (**Figures 5B, C**).

We finally sought to identify antigens commonly targeted across distinct states of Mtb control. Thus, IgG responses were interrogated across LTBI (where immune responses contain *Mtb* without progression to active disease), RSTRs (a unique state of controlled *Mtb* infection) and IV BCG immunized NHP (that experienced near sterilizing protection against *Mtb*) (**Figure 5C****).** Several antigens that were enriched in the IgG responses of different combinations of protected populations are shown in the overlapping regions of the Venn diagram (**Figure 5C**, **Supplementary Table 2**). Most strikingly, 36 antigens were preferentially targeted in all 3 protected populations (LTBI, RSTR, and IV BCG) as compared to the non-protected populations (ATB and ID BCG), as indicated in the center of the Venn diagram (unadjusted Mann-Whitney p-value < 0.1) (**Figure 5C**). 12 antigens at the center of the Venn diagram were significantly enriched in the “protected” groups following multiple testing correction including: Rv0583c (lpqN), Rv0915c (PPE14), Rv1142c (echA10), Rv2100, and Rv2721c (**Figure 5C**, **Supplementary Table 2**). Additional antigens enriched in the other combinations of protected populations were also identified (**Supplementary Table 2**). Collectively, these data reveal both antigens characteristic of disparate protected populations, as well as common antigens associated with control of TB that may represent antibody targets for next-generation therapeutic or TB vaccine design. Importantly, while certain antigens may be commonly targeted across datasets, direct integration or pooling of BAL and serum/plasma data may not fully capture compartment-specific immune biology.

### Validation of protein array antibody profiles

Protein microarrays synthesize and capture antigens or antigen segments using cell-free *in vitro* translation to generate proteome wide immunity, that may exhibit distinct post-translational profiles (58–60). However, whether these microarray signatures are correlated with traditional recombinant protein IgG binding titers and reflect differences in antibody functions that have been previously reported in the literature remains unclear. Thus, for a subset of antigens that showed differential reactivity across the human cohorts (**Supplementary Table 2**), we aimed to validate and extend humoral characterization by measuring protein binding antibody titers and functional antibody responses, using previously defined Systems Serology analysis (61) across ATB–LTBI groups (17) and RSTR–LTBI groups (7).

**Figure 6:**
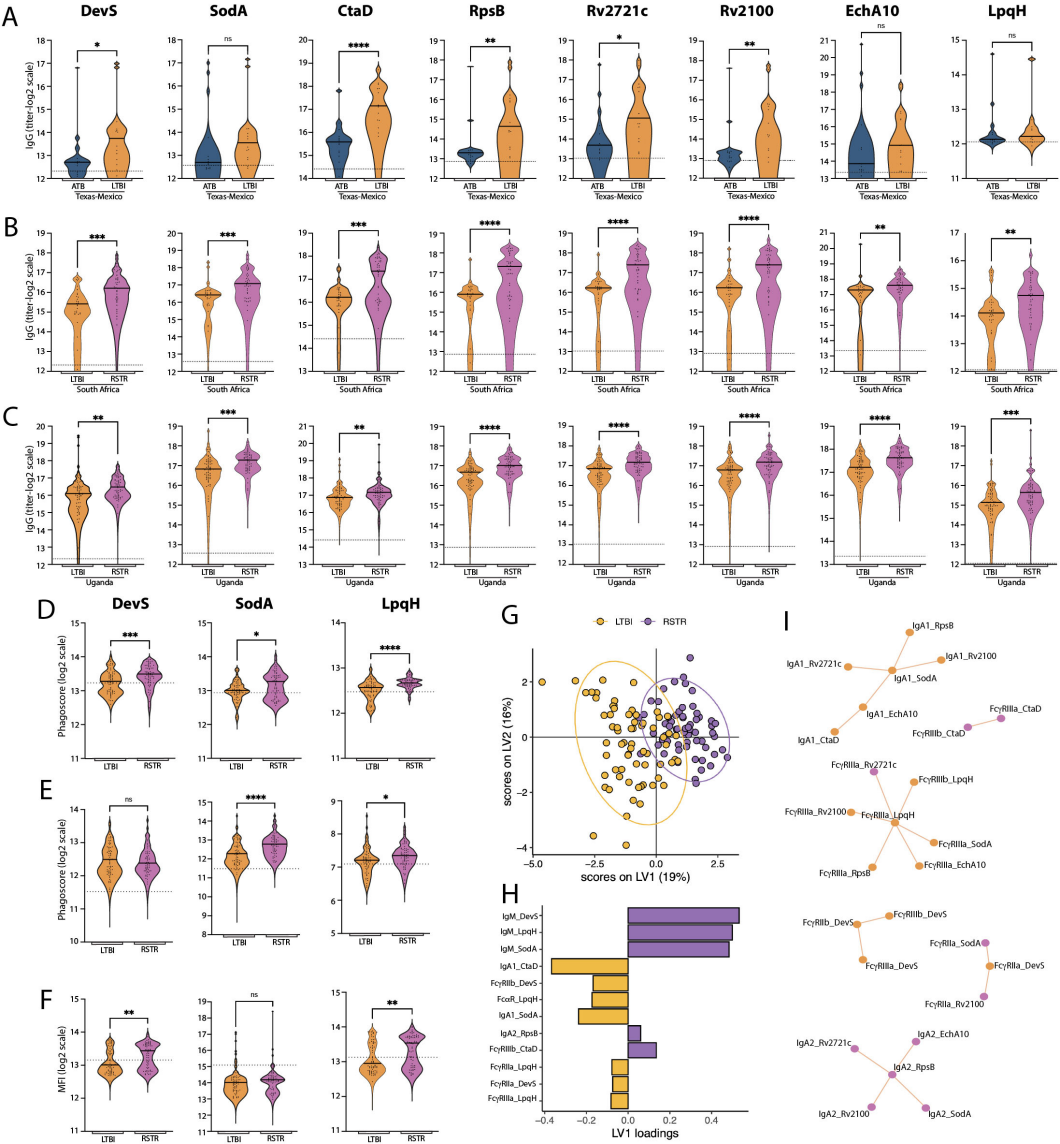
Differential IgG binding also tracks with functional differences across populations. **(A)** Violin plots show IgG titers in ATB (blue) and LTBI (yellow) serum samples collected from United States-Mexico border. **(B, C)** Violin plots show IgG titers in LTBI (yellow) and RSTR (purple) serum samples collected from Uganda **(B)** and South Africa **(C)**. **(D–F)** Violin plots demonstrate median antibody-dependent cellular phagocytosis (ADCP), **(D)**, antibody-dependent neutrophil phagocytosis (ADNP), **(E)** and antibody-dependent complement deposition (ADCD) across Ugandan RSTRs (purple) and LTBI (yellow). **(A–F)** Dashed lines denote IVIG titers. Solid black line represents the median for each group. Mann-Whitney p < 0.05 were considered significant. **(G)** LASSO PLS-DA analysis distinguish RSTR from LTBI. **(H)** Variable Importance in Projection (VIP) show the 12 antibody-features selected by the LASSO model to separate the 2 groups, including features in yellow enriched in LTBI and features in purple that are enriched in RSTRs along latent variable 1 (LV1). **(I)** The LASSO-correlation shows all the additional measured features that are co-correlated with the LASSO selected features that distinguish the 2 groups. * p< 0.05, ** p<<0.01, *** p< 0.001, **** p <0.0001, ns=non-significant Mann-Whitney U test.

In agreement with the proteome microarray data, IgG titers to recombinant Rv3132c (DevS), Rv3043c (CtaD), Rv2890c (RpsB), Rv2721c and Rv2100 were found to be significantly higher in the LTBI compared to ATB (**Figure 6A**). Similarly, elevated antibody titers were noted to all tested antigens among South African (**Figure 6B**) and Ugandan (**Figure 6C**) RSTRs compared to LTBI, highlighting similar profiles to those observed by microarray. These antigens were prioritized for downstream validation as they were available in-house at sufficient yield and quality; other candidates identified from the array were excluded due to challenges in expression following recombinant production.

Higher antibody titers to selected antigens may simply represent a marker of a unique immune response due to distinct microbial exposure or may actively contribute to enhanced antimicrobial control via enhanced antibody-mediated anti-microbial functions (62). To discern whether these uniquely targeted antigens were also associated with enhanced antibody functionality, we profiled the opsonophagocytic activity to a subset of antigens (available in larger quantities) was assessed in the Ugandan RSTR/LTBI groups. Rv3132c (DevS), Rv3846 (SodA) as well as Rv3763 (LpqH) specific antibodies exhibited enhanced monocyte phagocytosis in RSTR compared to LTBI (**Figure 6D****).** Similarly, Rv3846 (SodA)- and Rv3763 (LpqH)-antibodies from RSTR samples were also able to induce elevated neutrophil phagocytosis compared to LTBI (**Figure 6E**). Moreover, we also observed enhanced complement deposition by Rv3132c (DevS)- and Rv3763 (LpqH)-specific antibodies in RSTRs (**Figure 6F**), pointing to enhanced antibody functional activity in RSTRs to these unique antigens that could contribute to anti-microbial control. Ultimately, all systems serological data were combined for the Ugandan RSTR and LTBI individuals, including IgG subclasses, isotypes, and Fc-receptor (FcR) binding profiles and function, demonstrating significant separation in antibody profiles across the 2 groups using a LASSO/PLS-DA (**Figure 6G**), with a model accuracy of 81.9%. As few as 5-RSTR and 7-LTBI features, out of a total of 108 analyzed antibody features, were sufficient to separate groups (**Figure 6H**). Strikingly, significant differences in IgM, IgA, and FcR binding across the antigens drove the separation across the groups (**Figure 6H**), marked by a uniform enrichment of IgM to several antigens in RSTRs, along with Rv2890c (RpsB)-IgA2 levels and Rv3043c (CtaD)-FcγR3b binding responses. Conversely, the LTBI profile demonstrated an enrichment of IgA and FcαR binding antibodies and FcR binding responses to Rv3132c (DevS) and Rv3763 (LpqH) (**Figure 6H**). Given that the LASSO feature down selection reduces the number of features used to discriminate between groups, to avoid overfitting, we next examined the co-correlated antibody responses, to gain further insights into the unique overall humoral profiles across the groups. The LASSO correlate analyses pointed to an enrichment of mucosally relevant IgA2-responses (63) to all tested antigens in RSTRs, along with FcγR3a and b targeting to Rv3043 (CtaD) (**Figure 6I**). Conversely, an expanded network of cross-antigen IgA1, FcγR3a and FcγR2a binding was observed in the LTBI, with a smaller Rv3132c (DevS)-specific FcR network. Together, these results point to divergent cross-isotype and Fc-receptor binding profiles across RSTRs and LTBI, providing further insights into how these populations may functionalize particular antibody subpopulations in the response to *Mtb* exposure or infection.

### Overlap between antibody reactivity and dominant T cell antigens

Beyond inducing a potent humoral immune response, a highly efficacious TB vaccine will likely also elicit robust T cell immunity, given the critical role for T cell immunity in TB control (8–10). Hence, we next sought to identify antigens co-targeted by both antibody and T cell responses.

**Figure 7:**
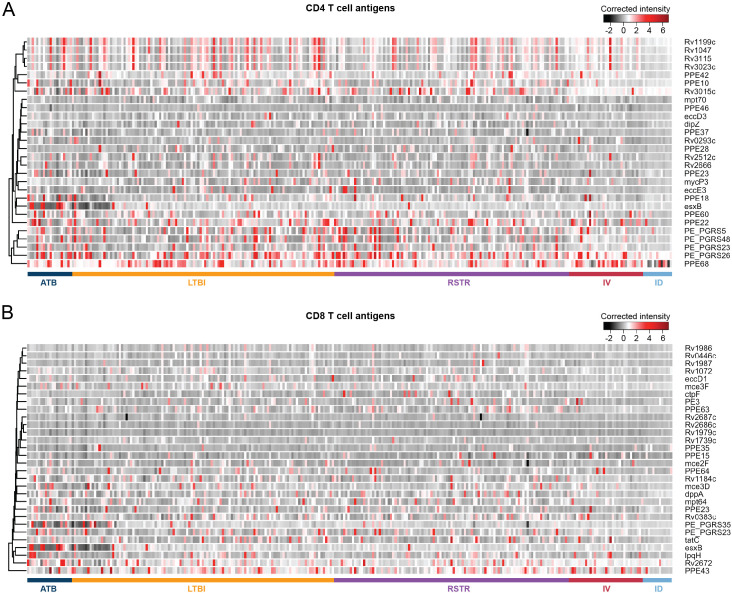
Overlap between antibody reactivity and dominant T cell antigens. **(A)** Heatmap showing batch-corrected IgG binding intensities to immunodominant **(A)** CD4 T cell antigens, and **(B)** CD8 T cell antigens, with detectable antibody reactivity in at least one population. Rows are hierarchically clustered by IgG reactivity pattern, and columns are ordered by cohort. Tile color indicates batch-corrected IgG binding intensity.

We first examined a set of 82 *Mtb* antigens previously found to account for approximately 80% of the CD4 T cell response in individuals with LTBI (64). Of note, the IgG binding intensity for these CD4 T cell antigens was significantly higher relative to the remainder of the proteome across all populations (**Supplementary Figure 8**). Despite this overall shift, individually, many of these antigens did not induce strong antibody responses, with only 31 of 82 exhibiting IgG reactivity above background in at least one population. Yet a subset of antigens were coordinately targeted by both CD4 T cell and antibody immunity (**Figure 7A**). These included antigens such as: Rv1047, Rv1441c (PE_PGRS26), Rv3873 (PPE68), and Rv3874 (EsxB). Rv3874 (EsxB) primarily elicited IgG responses in subjects with ATB, whereas the remaining antigens broadly elicited antibody responses across each of the studied populations (**Figure 7A**).

We next examined the overlap between CD8 T cell immunogenicity and antibody reactivity profiles across the study populations. Previous work identified a set of 74 immunodominant CD8 antigens in individuals with ATB and LTBI (65). In contrast to CD4-associated antigens, IgG binding intensities for CD8 T cell antigens showed only modest increases relative to the remainder of the proteome, reaching statistical significance in LTBI, RSTR, and ID BCG cohorts, but with small effect sizes (**Supplementary Figure 8**). Overall, 33 of these 74 antigens exhibited detectable antibody reactivity. Compared to the CD4 antigen set, IgG responses to CD8 antigens were generally weaker and observed in fewer individuals (**Figure 7B**), indicating less overlap between CD8 T cell and humoral antigenic targets. Nevertheless, a small subset of antigens such as, Rv0383c, Rv1980c (Mpt64), Rv2768c (PPE43), Rv3666c (DppA), and Rv3763 (LpqH) were found to elicit both potent CD8 T cell and humoral immune responses (**Figure 7B**). Collectively, these data identify a subset of *Mtb* antigens that are co-targeted by humoral and cell-mediated immunity, with greater overlap observed for CD4 than for CD8 T cell antigens.

## Discussion

Although *Mtb* remains the leading cause of death from a single infectious agent globally, efforts to develop effective vaccines that prevent infection or disease progression in adults have consistently failed—largely due to gaps in our understanding of the specific immune mechanisms that confer protection. While recent evidence supports roles for both T and B cells in immunity to *Mtb*, there is an urgent unmet need to identify and characterize novel *Mtb* antigen targets that are selectively targeted in the setting of control or clearance of *Mtb* infection. Such insights are essential to drive the development of next-generation vaccines, targeted therapeutics, and improved diagnostics. To begin to address this goal, we performed a proteome-wide analysis of *Mtb*-specific antibody responses across individuals representing a broad spectrum of TB disease states to determine whether distinct humoral signatures, as direct immune mediators or surrogates for T cells, are associated with differential clinical outcomes, thereby identifying novel candidate antigens for future vaccine, therapeutic, and diagnostic development. Across human and NHP populations vaccinated against and/or exposed to *Mtb*, much of the IgG response was directed towards surface-exposed antigens and secreted virulence factors. Discriminatory antigen-specific antibody responses were clearly observed across ATB and LTBI, RSTR and LTBI, as well as ID BCG and IV BCG immunized animals. Finally, an integrated analysis across cohorts identified a limited set of overlapping antigens targeted selectively in protective humoral immune responses including antigens that could drive *Mtb* control *in vivo*.

Emerging evidence increasingly supports a protective role for antibodies in the immune response against *Mtb*, challenging the long-standing view that T cells alone are responsible for mediating immunity (19, 66). Studies have shown that TB-specific antibodies exhibit distinct effector functions in individuals who control *Mtb* infection (7), antibodies have been implicated in protection induced by IV-BCG vaccination (42), TB-specific antibodies have been shown to restrict *Mtb* growth *in vitro* (18, 67), Fc-receptor knock out mice are more susceptible to TB disease (68), Fc-activity of *Mtb*-specific antibodies have been shown to be critical to their protective function (18, 69) and passive transfer experiments with both polyclonal and monoclonal antibodies have demonstrated protective effects in murine models of *Mtb* infection (21, 48, 51, 70–72). However, because antibody maturation and class switching are typically driven by T cell help, it is likely that protective immunity results from coordinated interactions between T and B cells. Recognizing this interdependence, we leveraged antigen-specific antibody responses as surrogates for either direct or indirect protection to systematically map novel *Mtb* antigens associated with immune control. Protein microarray technology offers a high-throughput approach to map whole proteome immunity to *Mtb* (25, 26, 73, 74). Despite *Mtb* peptide synthesis in a cell free IVTT system may not include post-translationally modified residues or conformational epitopes, antibody responses were detected to approximately 1267 antigens across our 5 populations, accounting for ~30% of the *Mtb* proteome. Moreover, responses were validated using recombinant proteins, demonstrating strong concordance with the array data. Interestingly, target protein pathway analysis, on proteins that were highly targeted in each sample set, pointed to enriched antibody responses to plasma membrane, cell wall, or secreted proteins, likely related to the abundance of many of these genes in the *Mtb* proteome involved in host immune evasion (26).

Pairwise comparisons of IgG binding profiles between exposure groups demonstrated that the different clinical populations could be accurately distinguished by IgG binding profiles using only a few antigens enriched in the antibody responses of each group, highlighting the potential for developing antibody-based diagnostics to disentangle differences in LTBI and ATB TB disease state. However, particularly in the human cohort comparisons, differences in antibody binding to individual antigens were often modest and did not remain significant following multiple testing correction. Moreover, while classification performance was robust, estimates derived through cross-validation may be optimistic compared to performance in independent or real-world diagnostic settings, particularly given the modest cohort sizes. Future studies in larger, independent cohorts will be important to validate and refine these antigen signatures for potential diagnostic applications.

In addition, this work considerably expands our understanding *Mtb*-proteome wide immunity by including antibody profiling of RSTRs whose immune profile may inform a distinct state of *Mtb* exposure (2), resulting in incomplete conversion of the traditional IFN-γ diagnostic tests in the absence of TB progression, as well as extending to IV BCG immunized rhesus macaques that displayed near sterilizing protection against *Mtb* (41). It is entirely possible that *Mtb* control is mediated by distinct immunological mechanisms across all these populations. Importantly, antibody responses measured in plasma and BAL represent distinct immunological compartments, and features such as antigen specificity, magnitude, and Fc characteristics may differ between systemic and mucosal responses. Thus, while the presence of an overlapping set of antigens that are targeted across RSTRs and IV BCG immunized NHP does not imply biological equivalence between compartments, it does points to the possibility of a common immunological mechanism, potentially driven by both T and B cells, that if induced appropriately, could lead to control of *Mtb*. Collectively, this *Mtb*-proteome IgG atlas offers an opportunity to define antibody reactivities that may be associated with different mechanisms of *Mtb* control.

A number of intriguing *Mtb* proteins and protein families emerged from these analyses that remain functionally unexplored, from the humoral immune perspective. For instance, PE and PPE proteins are two protein families comprising approximately 7% of the *Mtb* genome (40, 75). While the precise function of each remains elusive, evidence indicates that PE/PPE proteins are often secreted or cell surface associated and promote immune evasion and bacterial virulence (40). IgG binding was observed to PE/PPE proteins within each of the cohorts, and while antibody cross-reactivity to several of these proteins may exist due to the high amount of homology within the protein families (40), dissectible using orthogonal ELISA or Luminex, interesting heterogeneity in IgG responses to these proteins emerged across populations. Notably, PE/PPE proteins have also been reported to be enriched among immunodominant CD4 T cell antigens (64, 76), suggesting that this protein family may represent an important point of overlap between humoral and cellular antigen recognition. Yet, across the PE family, divided into PE and PE_PGRS subfamilies (40), both RSTRs and IV BCG immunized macaques elicited antibodies to selective PGRS subfamily members (39), suggesting a convergent focus of IgG humoral activity towards this unique subclass of proteins. Conversely, the absence of responses to ESAT-6 and Ag85b, that are dominantly targeted by T cell responses (77, 78), could reflect protein-limitations in the array technology, or future work could determine whether these results reflect differences in T and B cell immunodominance profiles to these antigens.

Although this study focused primarily on proteome-wide IgG profiling as a durable marker of antigen-specific humoral immunity and potential T cell help, the systems serology analysis revealed that additional antibody isotypes may provide important discriminatory information. In particular, IgM responses to several antigens contributed strongly to the separation of RSTR and LTBI subjects in the LASSO/PLS-DA model, suggesting that antigen-specific IgM may capture distinct features of *Mtb* exposure or immune control that are not fully reflected by IgG alone. These findings support the inclusion of IgM, along with IgA, IgG subclasses, Fc-receptor binding, and functional antibody readouts, in future studies aimed at defining humoral correlates of *Mtb* control. However, because IgM was assessed only in the targeted systems serology validation panel rather than in the initial proteome-wide discovery screen, these data should be interpreted as evidence that IgM is a promising complementary metric, rather than definitive evidence that IgM is superior to IgG for antigen discovery.

Several *Mtb* vaccines are currently being evaluated (79), including multi-valent mRNA-based TB vaccines, such as BNT164a1 and BNT164b1 (ClinicalTrials.gov Identifier: NCT05547464). However, mounting data points to the potential importance of additional antigens in *Mtb* control. Thus, efforts are urgently needed not only to define novel canonical and non-canonical T/B antigens, but also to test these antigens in relevant models to accelerate the development of an effective vaccines or therapeutics to curb the morbidity and mortality caused by TB. While significant work is still needed to fully realize the potential diagnostic and vaccine potential of the current study, this work defines an atlas of *Mtb*-targeting IgG responses across the TB disease spectrum which may inform protective immunity and guide subsequent antibody-based interventions to combat the TB epidemic.

## Materials and methods

### ATB-LTBI cohort

Sera utilized to probe proteome microarrays were from twenty adult HIV seronegative individuals with LTBI and twenty with ATB recruited from the south Texas (USA)/northeastern Mexico regions as described previously (28). LTBI was defined by the presence of positive TST and/or IFNγ-producing T cells specific for CFP10 and/or ESAT6 peptides with no previous history of TB diagnosis, treatment, or active clinical symptoms. ATB was defined by either positive sputum smear microscopy, positive culture for *Mtb*, and/or clinical diagnosis. All study participants gave written, informed consent. The study was approved by the institutional review boards of the participating institutions in the United States and Mexico.

### South African RSTR-LTBI cohort

A total of 30 LTBI subjects and 43 RSTRs were recruited from a study conducted in the South African goldmines at the Occupational Health Centre (OHC) in Orkney, North West Province between August 2015 and December 2016 as described previously (35, 36). Miners between the ages of 33 and 60 years old who had worked in the mining industry for at least 15 years were identified through pre-screening. Eligible subjects had no TB symptoms, no prior or current history of TB treatment, no silicosis, and were HIV negative. In the first version of the protocol (subjects enrolled July 10^th^, 2015 – October 29^th^, 2015), participants gave blood samples for QFT-GIT at enrollment and were followed up at 90 days where QFT-GIT was repeated and a TST was administered. In the second, simplified version of the protocol (subjects enrolled after October 20^th^, 2015), participants gave blood samples for QFT-GIT at enrollment and a TST was administered either at enrollment or one week later. All participants were followed up to 12 months later for additional QFT-GIT and TST administration. Subjects that maintained persistently negative QFT-GIT/TST results were defined as RSTRs. Subjects that tested QFT-GIT/TST positive were defined as LTBI subjects. All study participants gave written, informed consent. The study received ethical clearance from the participating institutions including the University of Witwatersrand Human Research Ethics Committee and the North West Health Research and Ethics Committee.

### Ugandan RSTR-LTBI cohort

A total of 129 Ugandans – 67 LTBI and 62 RSTRs – recruited from the Kawempe Community Health Study described previously (4, 5, 7, 80), were included in the present study. In brief, index individuals with pulmonary TB were identified by culture at the Uganda National Referral Tuberculosis Treatment Center at Upper Mulago Hospital in Kampala, Uganda. Household contacts of these index cases were then enrolled and followed prospectively for up to 2 years, where they were subjected to serial TST and clinical assessment for ATB at 0, 3, 6, 12, 18 and 24 months. 10.7% (n = 198) of all household contacts were TST-negative at the initial visit and remained TST-negative over the 2 years of follow-up. A subset of TST-negative (n = 162) and TST-positive LTBI subjects (n = 486) matched by age, and household or epidemiological risk score, were then reevaluated in a re-tracing study (4, 7, 81, 82). Reevaluation for *Mtb* infection status included three QFT assays – the first at the time of enrollment in the re-tracing study and the next two over the following 2 years – and a TST following the last QFT. Individuals that tested positive across 2 TSTs (one during the original TB contact study and one at the end of the re-tracing study) and 3 QFTs (during the re-tracing study) were categorized as LTBI individuals (n = 195). Individuals that tested negative across the same five assays were categorized as RSTRs (n = 82). Serum from subset of these HIV seronegative LTBI and RSTR subjects were used in the present study. All study participants gave written, informed consent. The study was approved by the institutional review boards of the participating institutions.

### Non-human primate BCG vaccination cohort

Rhesus macaque (*Macaca mulatta*) BAL samples used in the present study were collected during two studies previously performed at the Vaccine Research Center at the National Institutes of Health. In the first study, animals were immunized with BCG through different routes and doses^37^. Samples from 13 animals receiving standard ID BCG vaccination (target dose: 5 × 10^5^ CFUs) and 13 animals receiving IV BCG vaccination (target dose: 5 × 10^7^ CFUs) were included in the present study. BAL samples from 4 weeks post-BCG vaccination were analyzed. The second study included 20 additional rhesus macaques that received IV BCG vaccination (target dose: 5 × 10^7^ CFUs). BAL samples from these animals were analyzed between 2- and 5-weeks post-BCG vaccination. All experimentation and sample collection from both studies complied with ethical regulations at the respective institutions.

### Protein microarray fabrication and hybridization

*Mtb* proteome microarrays were produced and probed with sample using an IVTT in *Escherichia coli* as described previously (25, 26, 59). Across cohorts, successive versions of the *Mtb* protein microarray were used, reflecting incremental expansion of antigen coverage. In brief, open reading frames (ORFs) from the *Mtb* H37Rv genome were PCR-amplified using gene-specific primers. Large genes (>3 kb) were amplified in overlapping fragments. PCR products were cloned into the linearized T7 expression vector pXI via *in vivo* recombination cloning. Each plasmid was purified individually and used as a template for IVTT-based protein expression in E. coli. The translated protein products were printed onto nitrocellulose-coated Avid slides using an OmniGrid Accent microarray printer. Each subarray also contained negative control spots comprising IVTT reaction mixtures lacking DNA templates.

Prior to hybridization, slides were blocked with Surmodics Assay Diluent (“SmAD”, Cat#SM01-1000, Surmodics, Eden Prairie, MN) and serum or BAL samples were pre-absorbed with a 20% DH5α *E. coli* lysate solution in SmAD. Serum samples were diluted to a final concentration of 1:100 and BAL samples to 1:10 and were then incubated on the arrays overnight at 4 °C with gentle agitation. Following three washes in Tris-buffered saline containing 0.05% Tween-20 (TBS-T), bound IgG antibodies were detected using DyLight650-conjugated anti-human IgG secondary antibodies (Bethyl Cat#A80-104D5, Fortis Life Sciences, Boston, MA) diluted 1:250 in SmAD for both human and NHP samples. After three additional washes, slides were dried and scanned on a GenePix microarray scanner (Molecular Devices, San Diego, CA) for human samples and an InnoScan 910AL microarray scanner (Innopsis, Carbonne, France) for NHP samples.

TIFF images of each slide scan were exported from the microarray scanners, and fluorescence signal intensities were quantified using MAPIX 8.5 software (Innopsis, Carbonne, France) by calculating the median foreground minus median local background fluorescence for each spot. Quality control measures included exclusion of missing spots, contamination, or abnormal background variation. The final dataset was generated by normalizing intensity values as log_2_[(antigen-specific signal)/(median IVTT background)]. Antigens were defined as reactive when their normalized intensity exceeded log_2_(1.25), corresponding to approximately 1.25-fold above the sample’s median background control.

### Differential reactivity analysis

Mann-Whitney U tests were performed to assess differential IgG binding intensity to each antigen. The Benjamini-Hochberg procedure was used to correct for multiple hypothesis testing. In serum antibody comparisons of the ATB-LTBI both RSTR-LTBI cohorts, antigens with |μ_difference_| > 0.25 and an unadjusted Mann-Whitney p-value < 0.1 were classified as differentially reactive. For the BAL antibody comparison in the rhesus macaque BCG vaccination cohort, antigens with |μ_difference_| > 0.5 and an adjusted p-value < 0.1 were classified as differentially reactive. The Benjamini-Hochberg procedure was used to correct for multiple hypothesis testing in the rhesus macaque BCG vaccination cohort. Proteins with an antigen-specific signal less than 1.25-fold over the IVTT only background (normalized intensity < log_2_(1.25)) were considered below the limit of detection and not included in differential reactivity analysis. Statistical analyses were performed in R (version 4.1.1).

### Gene ontology enrichment analysis

For each antigen in the array, the fraction (percentage) of serum samples that displayed a normalized intensity score 1.25-fold over the IVTT only background was determined. The subset of antigens with a normalized intensity score 1.25-fold over background in over 75% of individuals in the study population were then used as the gene list input for GO enrichment analysis^65^, in which the GO cellular component terms over- or under-represented in the gene list were determined using a Fisher’s exact test. GO terms with Benjamini-Hochberg adjusted p-values < 0.1 were considered significant.

### Protein set enrichment analysis

Protein set enrichment analysis was used evaluate the protein classes enriched in the IgG response patterns of different clinical populations, as is commonly performed during gene set enrichment analysis of transcriptional data (33, 34). For each pairwise comparison (ATB vs. LTBI, RSTR vs. LTBI, or ID BCG vs. IV BCG) each protein in the array was ranked according to the following score:


$$protein\;significance\;score=(m_{B}-m_{A})*(-log_{10}(pval))$$

Where *m_A_* is the median normalized intensity score in Group A, *m_B_* is the median normalized intensity score in Group B, and *pval* is the p-value resulting from a Mann-Whitney comparison of group medians. This score was used to integrate statistical significance and magnitude difference into a single metric for better protein ranking. After ranking each protein in the array, enrichment analysis was performed using the fgsea package (version 1.20.0) in R (version 4.1.1) (33), to determine the extent to which particular protein sets were overrepresented at the top or bottom of the ranked list of proteins. *Mtb* gene sets used are from the Wellcome Sanger Institute functional annotations, which are available in the supplemental information.

### Partial least-squares discriminant analysis

A multivariate approach using a combination of LASSO feature selection and PLS-DA was utilized to identify antigens that differentiated the groups as described previously^38^. For analyses where independent cohorts were available (RSTR/LTBI), models were trained in one cohort (South Africa) and evaluated in an independent cohort (Uganda) to assess generalizability. For all other comparisons, model training, feature selection, and performance evaluation were conducted within a repeated cross-validation framework. In brief, bootstrap-resampled datasets were iteratively generated, and LASSO models were fit to each dataset, with the regularization parameter selected by internal cross-validation (31). Feature importance was quantified using variable inclusion probability, defined as the proportion of models in which the feature coefficient was non-zero. PLS-DA models across a range of variable inclusion probability thresholds were then evaluated using repeated cross-validation (32), in which data were repeatedly partitioned into training and test sets (5-fold cross validation repeated 100 times). The final model was selected based on the optimal trade-off between performance and interpretability. The first two latent variables (LVs) of the final model were plotted to visualize group separation. This learning algorithm was implemented using the glmnet package (version 4.1-3), and the mixOmics package (version 6.18.1) (83), in R (version 4.1.1).

### Multi-cohort integration

Normalized IgG binding intensity values from the four cohorts (ATB/LTBI, South African RSTR/LTBI, Ugandan RSTR/LTBI, and IDBCG/IVBCG) were combined for integrated analyses. To ensure comparability across datasets generated using successive versions of the *Mtb* protein microarray, analyses were first restricted to antigens assayed across all cohorts. From this shared set, proteins with an antigen-specific signal greater than 1.25-fold over the IVTT only background (normalized intensity < log_2_(1.25)) in at least one population were considered above the limit of detection (n=1,267) and kept in the combined dataset. No imputation of missing antigen measurements was performed. Principal component analysis (PCA) was used to initially visualize batch effects, and batch effects were subsequently removed through linear modeling (57). PLS-DA was used to project the data into a low dimensional space, where the first two LVs of the model were plotted to visualize group separation. This approached utilized the factoextra package (1.0.7), the limma package (version 3.50.1) (57)and the mixOmics package (version 6.18.1) (83), in R (version 4.1.1).

### Scatter plot for convergent IgG responses

Antigen reactivity was compared across BCG IV and RSTR groups by ranking antigens based on their median reactivity within each group. Reactivity was defined as the median response across subjects in each group. The RSTR group consisted of two datasets (South Africa, Uganda), the median reactivity values were first calculated within each dataset and then averaged to obtain a single reactivity score per group. A combined ranking was then determined across the groups. The top 100 antigens, based on this combined rank, were visualized in a scatter plot, with the top 7 antigens labeled for emphasis.

### Venn diagram

For the outer regions of the Venn diagram, Mann-Whitney U tests were performed to assess differential IgG binding intensity to each antigen using a “one versus all” approach. For instance, antigens present in the IV BCG section of the Venn diagram had a higher IgG binding intensity (unadjusted Mann-Whitney p-value < 0.1) in IV BCG subjects than in subjects spanning the remaining *Mtb*-exposure and vaccination groups (ATB, LTBI, RSTR, and ID BCG). In the overlapping regions of the Venn diagram (e.g. IV BCG ∩ RSTR), the same statistical approach was taken such that antigens present in the overlapping region were those with a higher IgG binding intensity (unadjusted Mann-Whitney p-value < 0.1) in the overlapping groups (in this case: IV BCG and RSTR) than the subjects spanning the remaining groups (in this case: ATB, LTBI, and ID BCG). Hence, antigens present in the center of the Venn diagram exhibited a higher IgG binding intensity (unadjusted Mann-Whitney p-value < 0.1) in “protected” groups (LTBI, RSTR, and IV BCG) as compared to the “unprotected groups” (ATB and ID BCG). Bolded antigens indicate those that were significant following multiple testing correction by the Benjamini-Hochberg procedure. Batch corrected IgG binding intensity values were used. Statistical analyses were performed in R (version 4.1.1).

### Recombinant protein production

Gene blocks for *ctaD*, *rpsB, Rv2721c, Rv2100* and *echA10* cloned in pET-28b (+) were custom ordered. The expression plasmids were transformed in One shot BL21 Star (DE3) Chemically competent *E. coli* (ThermoFischer Scientific) and selected on LB- Kanamycin (50 μg/mL) agar plates. Recombinant proteins were purified as mentioned previously (84). Briefly, 10 ml of overnight grown primary culture in LB with Kanamycin (50 μg/mL) were diluted 1:100 into 300 ml of LB with Kanamycin (50 μg/mL) and grown at 37 °C till OD_600_s reached 0.4-0.5. Protein expression was induced with 0.1 mM isopropyl-D-thiogalactopyranoside at 37 °C for 3 h or at 22 °C for 16h. Cells were resuspended in lysis buffer [20 mM sodium phosphate, (pH 7.4), 50 mM NaCl, 5% glycerol, 1 mM DTT] with 1 mM phenylmethylsulfonylfluoride (PMSF) and disrupted via sonication. Cell debris was clarified, and supernatants were mixed with pre-equilibrated Ni^2+^-NTA Beads (Sigma) at 4 °C overnight. Protein bound to beads were washed with 5-bed volumes of wash buffer (20 mM sodium phosphate, (pH 7.4), 200 mM NaCl and 5 mM imidazole) and were eluted in 2 ml elution buffer [20 mM sodium phosphate, (pH 7.4), 50 mM NaCl, 1 mM DTT, 300 mM imidazole]. Peak fractions were dialyzed using Slide-A-Lyzer (Pierce) against dialysis buffer (20 mM sodium phosphate, (pH 7.4), and 20% glycerol) and concentrated using 3kD MWCO Amicon Ultra Centrifuge Filter. The final protein concentration was measured using absorbance at 280 nm, normalized, and confirmed by Coomassie-stained polyacrylamide gels against standard BSA stocks. DevS and SodA were kindly provided by Tom Ottenhoff and LpqH was purchased from BEI Resources (NR-50757).

### Luminex

*Antigen*-specific antibodies of various isotypes and Fc-Receptor binding differences were determined using a customized Luminex assay, as previously described (85). Carboxylated magplex-microspheres (Luminex Corp) of distinct bead regions were coupled to individual protein antigens by carbodiimide-NHS ester-coupling using by EDC and NHS (Thermo Fisher), as previously described (86). A master mix of antigen-coupled beads were prepared in 0.1%BSA-PBS and incubated with diluted serum samples in duplicates (1:60 for Total IgG, IgG1, IgG3, IgA1, IgA2, all Fc-receptors and 1:30 for IgG2, IgG4, IgM) overnight at 4 °C in clear flat-bottom 384-well plates (Greiner Bio-One), shaking at 700 rpm. On the following day, plates were washed using an automated magnetic plate washer. PE-coupled mouse anti-human antibodies (Southern Biotech) were added to each well and the plates were incubated at room temperature for one hour, shaking at 700 rpm. To detect FcR binding, Fc-receptor were coupled with PE, as mentioned previously (85) and were added to the immune complexes. Plates were washed and immune complexes were resuspended in Q-Sol buffer (Sartorius). Fluorescence was acquired on an Intellicyt iQue, and PE median fluorescent intensity (MFI) was reported to assess relative antigen-specific antibody titers and FcR binding. Intravenous Immunoglobulins (IVIG) from Sigma, Flebogamma, Innovative and Gammagard were used to define a baseline.

### Antibody-dependent cellular phagocytosis

Biotinylated antigens were attached to fluorescent yellow-green neutravidin microspheres (Invitrogen), as described previously (7). In a tissue culture round bottom 96 well plate, 10 µl of antigen-coated beads were incubated with 10 µl of 1:60 diluted serum samples in duplicates at 37 °C for 2 h to form immune complexes. Immune complexes were centrifuged at 2000 g for 10 min and supernatant was discarded. THP-1 cells (25000 per well) were added to the immune complexes and incubated at 37 °C, 5% CO_2_ for 16 h. Following day, cells were washed, fixed with 4% paraformaldehyde and resuspended in PBS. Bead uptake was quantified on Intellicyt iQue (Sartorius). Phagocytic scores represent: ((% FITC-positive cells) × (geometric mean fluorescence intensity of the FITC-positive cells)) divided by 10,000.

### Antibody-dependent neutrophil phagocytosis

Biotinylated antigens were attached to fluorescent red neutravidin microspheres (Invitrogen). In a tissue culture round bottom 96 well plate, 10 µl of antigen-coated beads were incubated with 10 µl of 1:60 diluted serum samples in duplicates at 37 °C for 2 h to form immune complexes. Fresh peripheral blood from healthy donors collected in ACD anticoagulant tubes were mixed with ACK lysis buffer (Thermo Fischer) at a 1:10 ratio and incubated for 5 min at RT with intermittent invert mixing to lyse red blood cells. Cells was centrifuged for 5 min at 1500 rpm and cell pellet containing leukocytes were washed with PBS and resuspended in complete RPMI (Sigma) containing 10% fetal bovine serum (Sigma), 10 mM HEPES (Corning) and 2 mM L-glutamine (Corning). Leukocytes (50000 per well) were added to the immune complexes and incubated at 37 °C, 5% CO_2_ for 1 h. Post incubation, cells were washed, stained with antihuman Pacific Blue CD66b antibody (BioLegend), fixed and resuspended in PBS. Florescence was acquired on Intellicyt iQue (Sartorius). Phagocytic scores were calculated only in the CD66b-positive cell population.

### Antibody-dependent complement deposition

Carboxylated magplex-microspheres (Luminex Corp) of distinct bead regions were coupled to individual protein antigens by carbodiimide-NHS ester-coupling using by EDC and NHS (Thermo Fisher). To form immune complexes, antigen-coupled beads were incubated with 5 µl of 1:30 diluted serum samples in duplicates in a 384 well plate at room temperature for 2 h, shaking at 700 rpm. Plates were washed and incubated with lyophilized guinea pig complement (Cedarlane) diluted in gelatin veronal buffer with calcium and magnesium (Boston BioProducts) at room temperature shaking at 700 rpm. Post 20 mins incubation, plates were washed twice with PBS containing 15mM EDTA and Fluorescein-Conjugated Goat IgG Fraction to Guinea Pig Complement C3 (Mpbio) was added. Following incubation at room temperature for 30 min, plates were washed twice with 0.1%BSA in PBS and immune complexes were resuspended in PBS. Florescence was acquired on Intellicyt iQue (Sartorius) and median fluorescent intensity (MFI) was reported as a readout.

## Data Availability

The original contributions presented in the study are included in the article/**Supplementary Material**. Further inquiries can be directed to the corresponding author.

## Supplementary material

Supplementary Figure 1 Summary of the cohorts utilized for Mtb proteome-wide IgG profiling. Four distinct cohorts were analyzed. Distinct clinical populations include: active tuberculosis (ATB); latent tuberculosis infection (LTBI); highly Mtb exposed, but uninfected resisters (RSTR); rhesus macaques immunized with intradermal BCG (ID BCG); rhesus macaques immunized with intravenous BCG (IV BCG). Created with BioRender.

Supplementary Figure 2 Protein class analysis from ATB-LTBI cohort. (**A**) Manhattan plot showing overall median intensity of each antigen. Proteins with an antigen-specific signal less than 1.25-fold over the IVTT only background in each group (normalized intensity < log_2_(1.25)) were considered below the threshold of detection (light blue shaded area). (**B**) Protein set enrichment analysis across groups using Sanger gene sets. Gene sets enriched in ATB are blue, gene sets enriched in LTBI are yellow. Points are labeled with the normalized enrichment score. Gene sets greater than the dashed line at a Benjamini-Hochberg adjusted p-value of 0.25 were considered significant.

Supplementary Figure 3 Additional analysis of South African RSTR-LTBI cohort. (**A**) Manhattan plot showing overall median intensity of each antigen. Proteins with an antigen-specific signal less than 1.25-fold over the IVTT only background in each group (normalized intensity < log_2_(1.25)) were considered below the threshold of detection (light blue shaded area). (**B**) LASSO PLS-DA analysis distinguishing RSTRs from LTBI subjects in the South Africa cohort by IgG binding profile. Score plot (left). Ellipses show 95% confidence intervals for each population. LV1 loadings plot of LASSO-selected antigens (right). (**C**) Protein set enrichment analysis across groups using Sanger gene sets. Gene sets enriched in RSTRs are purple, gene sets enriched in LTBI are yellow. Points are labeled with the normalized enrichment score. Gene sets greater than the dashed line at a Benjamini-Hochberg adjusted p-value of 0.25 were considered significant.

Supplementary Figure 4 Analysis of Uganda RSTR-LTBI cohort. (**A**) Volcano plot showing differential reactivity analysis in the Uganda RSTR-LTBI cohort. Antigens with |μ_LTBIintensity_ – μ_RSTRintensity_ | > 0.25 (vertical dashed lines) and unadjusted Mann-Whitney p-value < 0.1 (horizontal dashed line) were considered differentially reactive and are colored. RSTR-enriched antigens (purple). LTBI-enriched antigens (yellow). (**B** and **C**) LASSO PLS-DA analysis distinguishing RSTRs from LTBI subjects in the Ugandan cohort by IgG binding profile, (**B**) Score plot. Ellipses show 95% confidence intervals, (**C**) LV1 loadings plot of LASSO-selected antigens.

Supplementary Figure 5 Common RSTR and LTBI antigens. Normalized IgG binding intensity to the set of antigens consistently enriched in the IgG responses of (**A**) RSTRs, and (**B**) individuals with LTBI. South Africa RSTR-LTBI cohort is on the top and Uganda RSTR-LTBI cohort is on the bottom in each panel. Dashed line shows the background binding level (normalized intensity = log_2_(1.25)). Mann-Whitney, unadjusted p-values < 0.1 are shown.

Supplementary Figure 6 Additional rhesus BCG vaccination cohort analysis. Manhattan plot showing overall median intensity of each antigen. Proteins with an antigen-specific signal less than 1.25-fold over the IVTT only background in each group (normalized intensity < log_2_(1.25)) were considered below the threshold of detection (light blue shaded area).

Supplementary Figure 7 Batch correction of integrated dataset. Principal component analysis of the, (**A**) uncorrected and, (**B**) batch-corrected datasets. Batch 1 (ATB-LTBI); Batch 2 (South Africa and Uganda RSTR-LTBI); Batch 3 (IDBCG-IVBCG).

Supplementary Figure 8 IgG reactivity to dominant T cell antigens compared to the remainder of the Mtb proteome. Box plots showing batch-corrected IgG binding intensities to immunodominant CD4 T cell antigens, immunodominant CD8 T cell antigens, and all other antigens across each population. Unadjusted Mann-Whitney p-values are shown for comparisons between each T cell antigen set and all other antigens.

Supplementary Table 1 Demographic characteristics of human clinical cohorts included in the current study.
