# Supplementary figures and images for: A proteome-wide atlas of humoral immunity to Mycobacterium tuberculosis across the spectrum of disease
Source: Front Immunol. 2026 Jun 3;17:1810894. doi: 10.3389/fimmu.2026.1810894 (PMC13271977; doi:10.3389/fimmu.2026.1810894)

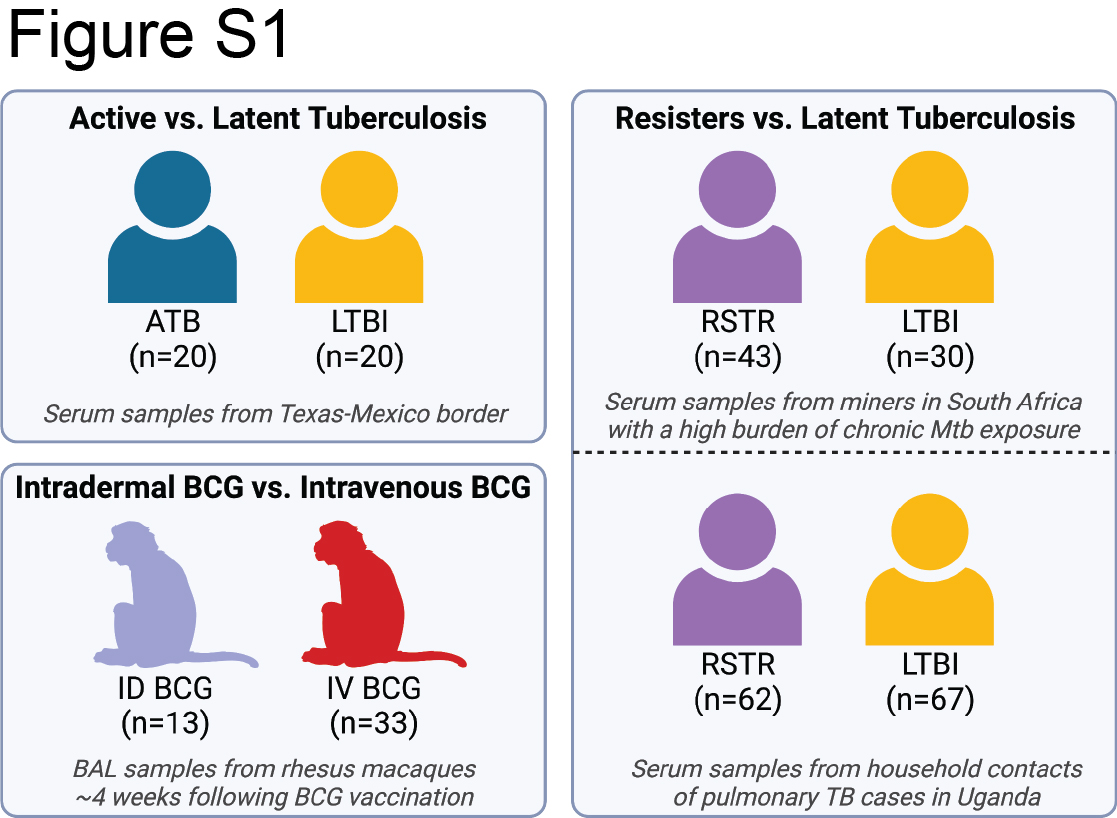

Supplement: Supplementary Figure 1 — Summary of the cohorts utilized for Mtb proteome-wide IgG profiling. Four distinct cohorts were analyzed. Distinct clinical populations include: active tuberculosis (ATB); latent tuberculosis infection (LTBI); highly Mtb exposed, but uninfected resisters (RSTR); rhesus macaques immunized with intradermal BCG (ID BCG); rhesus macaques immunized with intravenous BCG (IV BCG). Created with BioRender. [file Image1.jpeg]

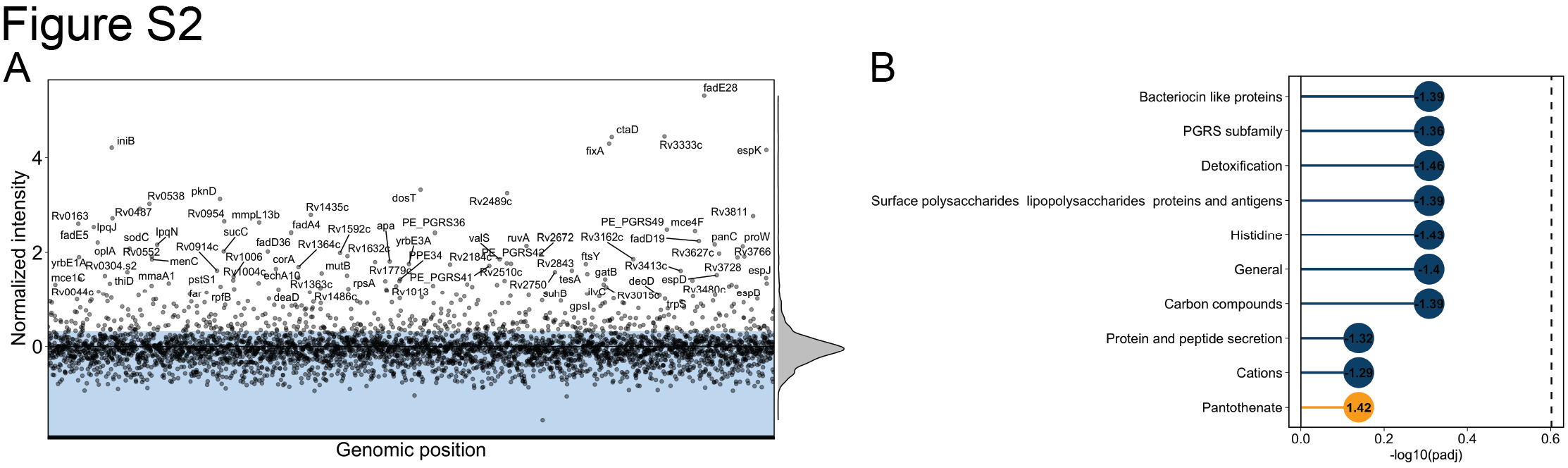

Supplement: Supplementary Figure 2 — Protein class analysis from ATB-LTBI cohort. (A) Manhattan plot showing overall median intensity of each antigen. Proteins with an antigen-specific signal less than 1.25-fold over the IVTT only background in each group (normalized intensity < log2(1.25)) were considered below the threshold of detection (light blue shaded area). (B) Protein set enrichment analysis across groups using Sanger gene sets. Gene sets enriched in ATB are blue, gene sets enriched in LTBI are yellow. Points are labeled with the normalized enrichment score. Gene sets greater than the dashed line at a Benjamini-Hochberg adjusted p-value of 0.25 were considered significant. [file Image2.jpeg]

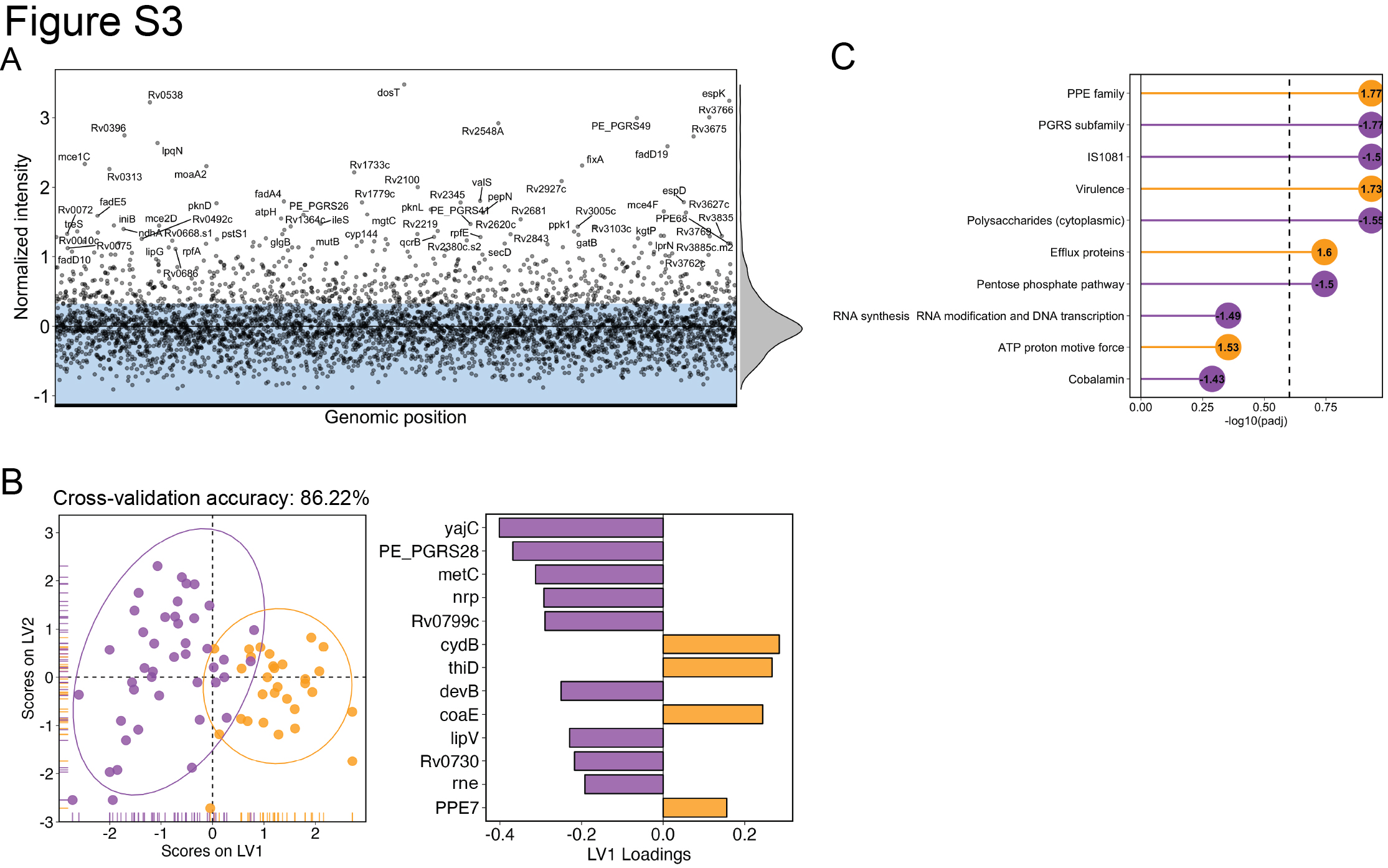

Supplement: Supplementary Figure 3 — Additional analysis of South African RSTR-LTBI cohort. (A) Manhattan plot showing overall median intensity of each antigen. Proteins with an antigen-specific signal less than 1.25-fold over the IVTT only background in each group (normalized intensity < log2(1.25)) were considered below the threshold of detection (light blue shaded area). (B) LASSO PLS-DA analysis distinguishing RSTRs from LTBI subjects in the South Africa cohort by IgG binding profile. Score plot (left). Ellipses show 95% confidence intervals for each population. LV1 loadings plot of LASSO-selected antigens (right). (C) Protein set enrichment analysis across groups using Sanger gene sets. Gene sets enriched in RSTRs are purple, gene sets enriched in LTBI are yellow. Points are labeled with the normalized enrichment score. Gene sets greater than the dashed line at a Benjamini-Hochberg adjusted p-value of 0.25 were considered significant. [file Image3.jpeg]

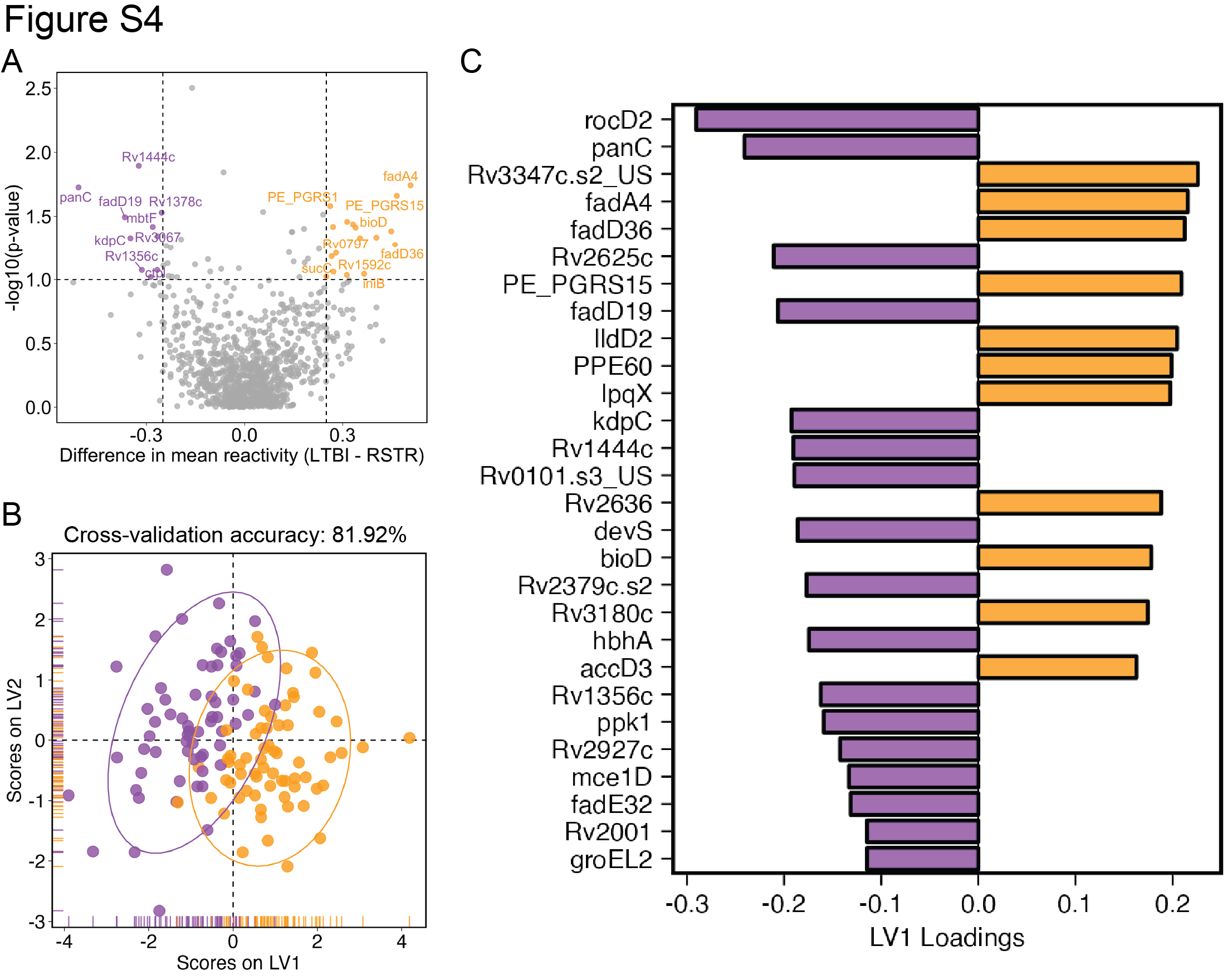

Supplement: Supplementary Figure 4 — Analysis of Uganda RSTR-LTBI cohort. (A) Volcano plot showing differential reactivity analysis in the Uganda RSTR-LTBI cohort. Antigens with |μLTBIintensity – μRSTRintensity | > 0.25 (vertical dashed lines) and unadjusted Mann-Whitney p-value < 0.1 (horizontal dashed line) were considered differentially reactive and are colored. RSTR-enriched antigens (purple). LTBI-enriched antigens (yellow). (B and C) LASSO PLS-DA analysis distinguishing RSTRs from LTBI subjects in the Ugandan cohort by IgG binding profile, (B) Score plot. Ellipses show 95% confidence intervals, (C) LV1 loadings plot of LASSO-selected antigens. [file Image4.jpeg]

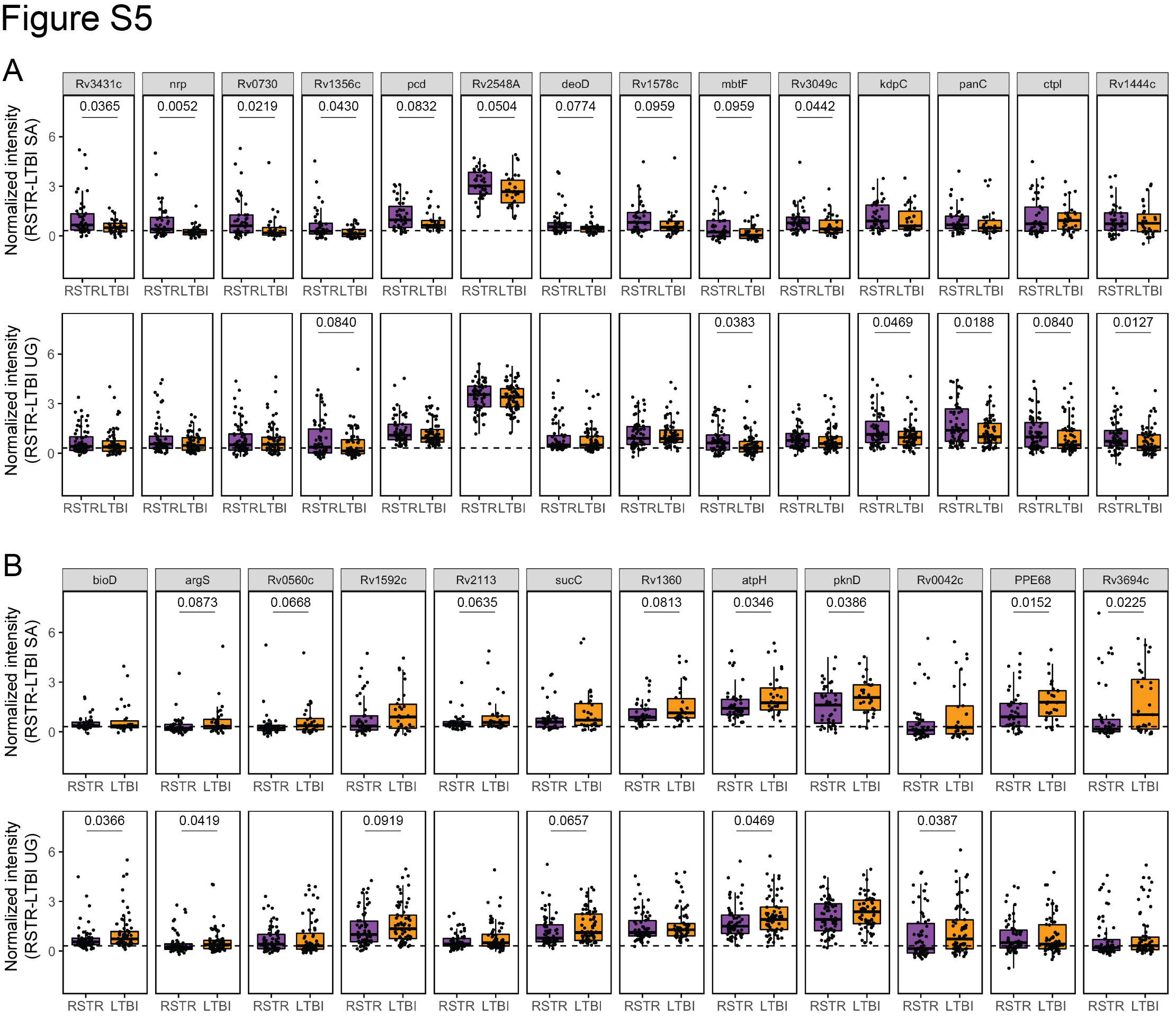

Supplement: Supplementary Figure 5 — Common RSTR and LTBI antigens. Normalized IgG binding intensity to the set of antigens consistently enriched in the IgG responses of (A) RSTRs, and (B) individuals with LTBI. South Africa RSTR-LTBI cohort is on the top and Uganda RSTR-LTBI cohort is on the bottom in each panel. Dashed line shows the background binding level (normalized intensity = log2(1.25)). Mann-Whitney, unadjusted p-values < 0.1 are shown. [file Image5.jpeg]

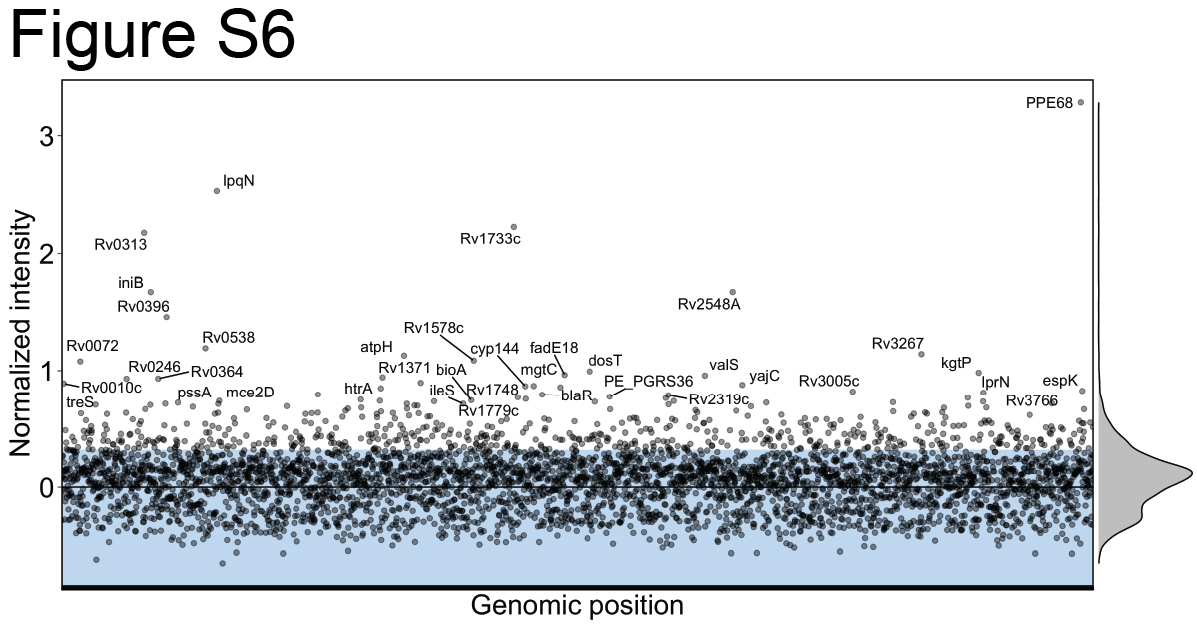

Supplement: Supplementary Figure 6 — Additional rhesus BCG vaccination cohort analysis. Manhattan plot showing overall median intensity of each antigen. Proteins with an antigen-specific signal less than 1.25-fold over the IVTT only background in each group (normalized intensity < log2(1.25)) were considered below the threshold of detection (light blue shaded area). [file Image6.jpeg]

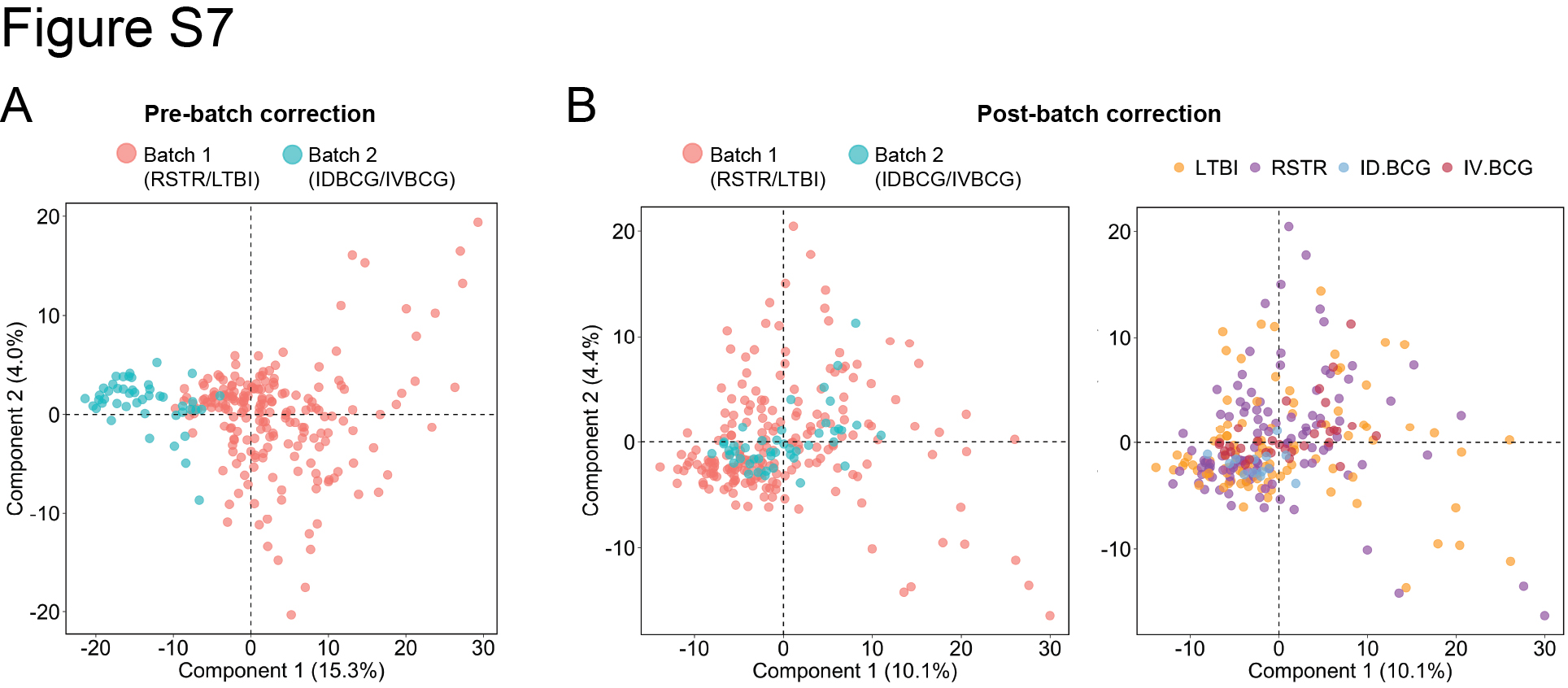

Supplement: Supplementary Figure 7 — Batch correction of integrated dataset. Principal component analysis of the, (A) uncorrected and, (B) batch-corrected datasets. Batch 1 (ATB-LTBI); Batch 2 (South Africa and Uganda RSTR-LTBI); Batch 3 (IDBCG-IVBCG). [file Image7.jpeg]

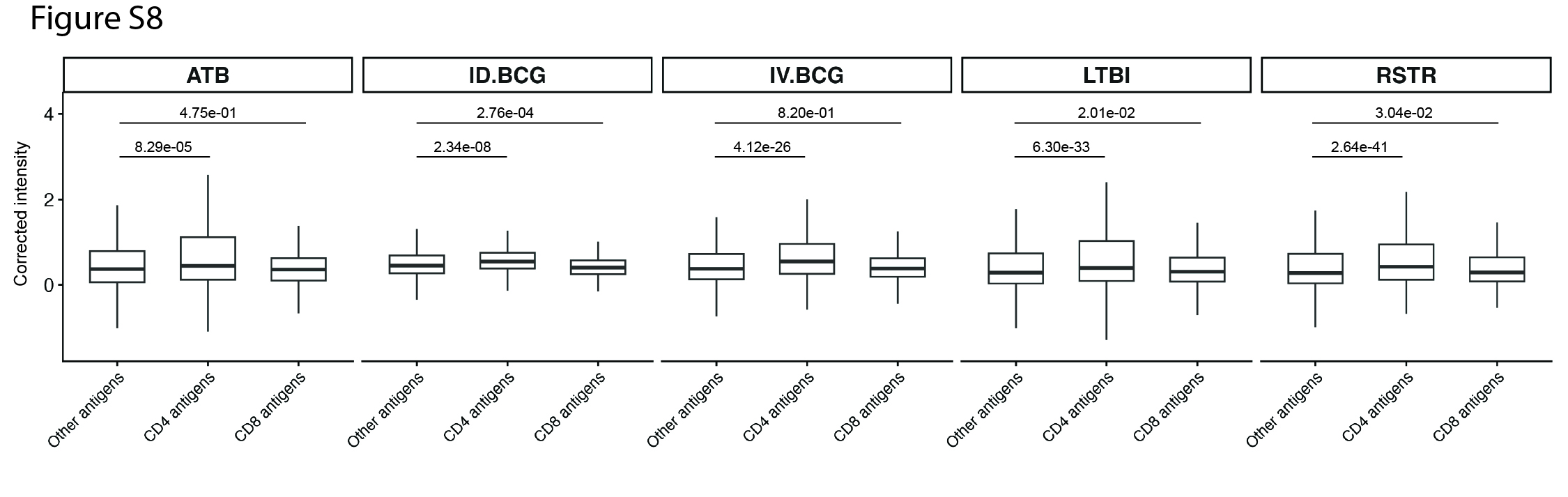

Supplement: Supplementary Figure 8 — IgG reactivity to dominant T cell antigens compared to the remainder of the Mtb proteome. Box plots showing batch-corrected IgG binding intensities to immunodominant CD4 T cell antigens, immunodominant CD8 T cell antigens, and all other antigens across each population. Unadjusted Mann-Whitney p-values are shown for comparisons between each T cell antigen set and all other antigens. [file Image8.jpeg]
